# PANoptosis‐Related Diagnostic Biomarkers in Non‐Neovascular Age‐Related Macular Degeneration: An Integrative Transcriptomic and Experimental Study

**DOI:** 10.1155/genr/8903808

**Published:** 2026-02-13

**Authors:** Jiaming Li, Yirong Ma, Miao Hu, Qian Zhang, Anqi Wang, Qiuyu Tang, Qinshang Guo, Binglin Huang

**Affiliations:** ^1^ Department of Postgraduate, Jiangxi University of Chinese Medicine, Nanchang, China, jxutcm.edu.cn; ^2^ Department of Clinical Medicine, Jiangxi University of Chinese Medicine, Nanchang, China, jxutcm.edu.cn

**Keywords:** age-related macular degeneration, experimental validation, machine learning, PANoptosis, single-cell transcriptomics

## Abstract

Age‐related macular degeneration (AMD), particularly its non‐neovascular (dry) form, is a progressive retinal disorder that causes central vision loss and substantial impairment in daily life. Inflammation and immune dysregulation are recognized as core drivers of AMD, yet the contribution of PANoptosis, a form of programmed cell death that integrates pyroptosis, apoptosis, and necroptosis, remains unclear. In this study, we integrated human single‐cell transcriptomic and bulk microarray datasets from the retina and retinal pigment epithelium–choroid to characterize PANoptosis‐related transcriptional changes in dry AMD. Dimensionality reduction, cell‐type annotation, and PANoptosis gene‐set scoring revealed a distinct PANoptosis signature enriched in AMD, with particularly strong activation in myeloid populations. By combining differential expression analysis with machine learning‐based feature selection, we identified four PANoptosis‐related genes (PON2, BNIP3, EPHB6, and TPD52) that robustly distinguished AMD from control samples and were associated with an altered immune microenvironment. Genetic instrument analysis further suggested a positive association between TPD52 expression and AMD risk. At the cellular level, our data highlighted macrophages, especially pro‐inflammatory M1‐like macrophages, as key coordinators of PANoptosis‐related pathways in dry AMD. To validate these findings in vivo, we used a sodium iodate‐induced mouse model of dry AMD and observed significant dysregulation of PON2, BNIP3, EPHB6, and TPD52 in the retina by RT‐qPCR, consistent with the human transcriptomic results and supporting their involvement in retinal degeneration and inflammation. Together, these findings implicate PANoptosis as an important and previously underappreciated component of dry AMD pathophysiology, define a four‐gene PANoptosis‐related signature with diagnostic potential, and suggest new molecular targets for therapeutic intervention.

## 1. Introduction

Age‐related macular degeneration (AMD) is a common retinal degenerative disease that stands as one of the primary causes of significant visual impairment or blindness among middle‐aged and older populations. In developed countries, AMD is the foremost cause of irreversible blindness among individuals aged 60 and above. The World Health Organization (WHO) identifies AMD as the third most prevalent cause of global vision impairment. It is anticipated that by 2040, the global number of individuals affected by AMD will reach 288 million, corresponding to a prevalence rate of 8.7% [1]. As the global population ages, the incidence of AMD continues to increase, placing a significant social and economic burden on society. AMD is principally categorized into two types: atrophic (dry) and exudative (wet). Dry AMD accounts for approximately 80%–90% of cases [2] and is characterized by the progressive atrophy of retinal pigment epithelium (RPE) cells and photoreceptor cells. In contrast, wet AMD is primarily characterized by choroidal neovascularization (CNV), often accompanied by hemorrhage, exudation, and fibrosis, leading to rapid progression and a high risk of blindness. In terms of treatment, antivascular endothelial growth factor (anti‐VEGF) therapy is the primary treatment for wet AMD and yields favorable results in the short term. However, long‐term follow‐up studies have shown that 71.9% of patients experience a decline in vision to pretreatment levels, or even worse, 5 years posttreatment [3]. Currently, no effective treatment exists for dry AMD, and research into early diagnostic biomarkers for AMD has progressed slowly, underscoring the urgent need for in‐depth exploration of its molecular mechanisms. In line with this unmet need, the present study focuses primarily on the dry AMD spectrum, including early/intermediate AMD and geographic atrophy.

The pathogenesis of AMD is complex, involving multiple biological processes, including oxidative stress [4], RPE cell senescence [5], immune‐inflammatory responses, endoplasmic reticulum stress, and autophagy [5, 6], as well as genetic susceptibility [7]. In recent years, the role of programmed cell death (PCD) in AMD development has garnered increasing attention. PCD encompasses various forms, such as apoptosis, pyroptosis, and necroptosis, all of which play significant roles in inflammation and immune regulation [8]. However, the mechanisms through which these forms of cell death contribute to the pathological processes of AMD remain unclear. Notably, PANoptosis, an emerging form of PCD, combines the characteristics of pyroptosis, apoptosis, and necroptosis. The occurrence of PANoptosis is regulated by the PANoptosome complex, which integrates key molecular components from all three cell death pathways. Studies have demonstrated that PANoptosis plays a crucial role in various inflammatory diseases and infections [9, 10], but its specific involvement in AMD progression requires further investigation.

This study aims to identify key genes and potential diagnostic biomarkers associated with PANoptosis in AMD by integrating single‐cell transcriptomic data, microarray data, and experimental validation. First, we used single‐cell transcriptomic data to perform detailed annotation and clustering analysis of retinal cells from AMD patients and healthy controls, identifying DEGs related to PANoptosis. Subsequently, Mendelian randomization (MR) analysis, in combination with various machine learning algorithms, was employed to further investigate the genetic underpinnings and refine the selection of key biomarkers (Figure 1).

FIGURE 1Workflow of the analysis. (a) scRNA‐seq and differential expression analysis. (b) SCENIC and cell‐to‐cell communications analysis. (c) Acquisition of shared genes and enrichment analysis. (d) MR and colocalization analysis. (e) Acquisition of core genes and GSEA/GSVA. (f) Construction of diagnostic models. (g) Experimental verification.

**Figure:**
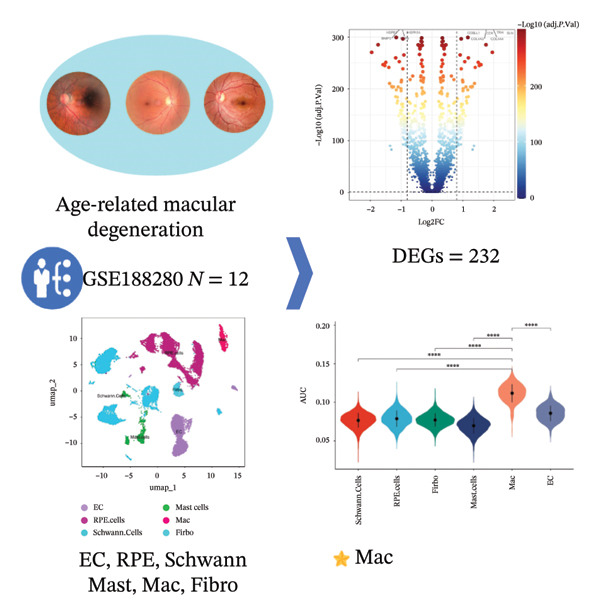
(a)

**Figure:**
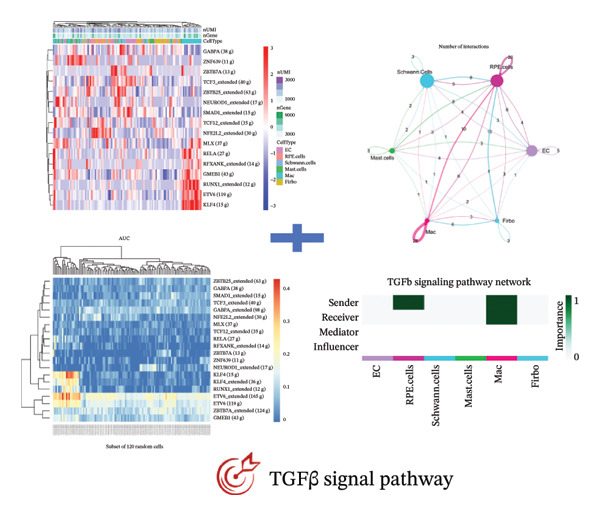
(b)

**Figure:**
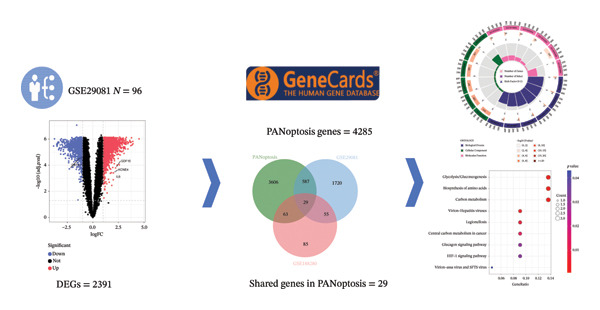
(c)

**Figure:**
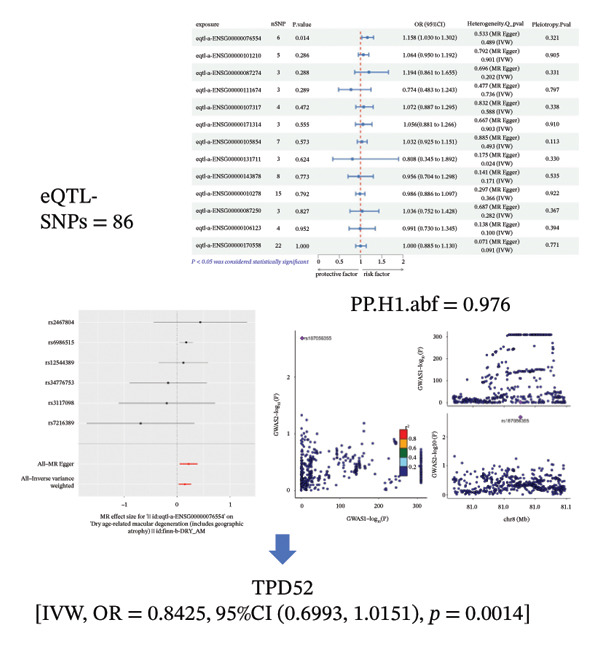
(d)

**Figure:**
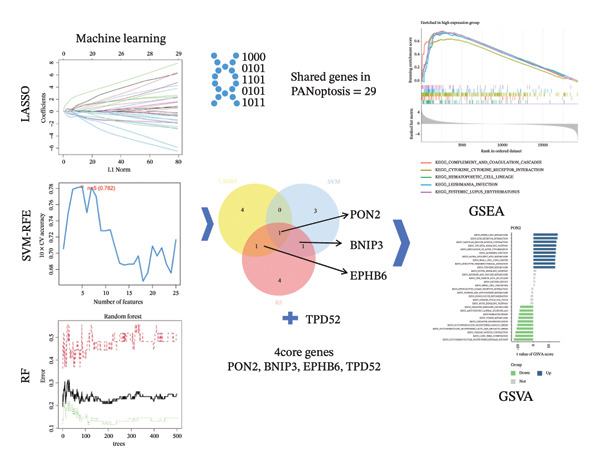
(e)

**Figure:**
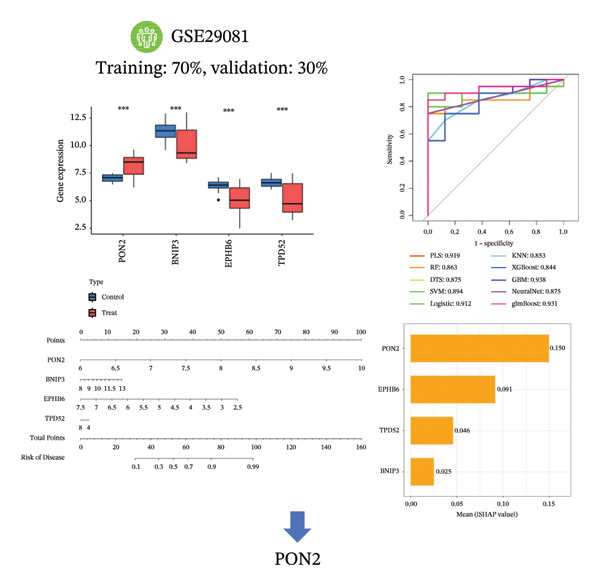
(f)

**Figure:**
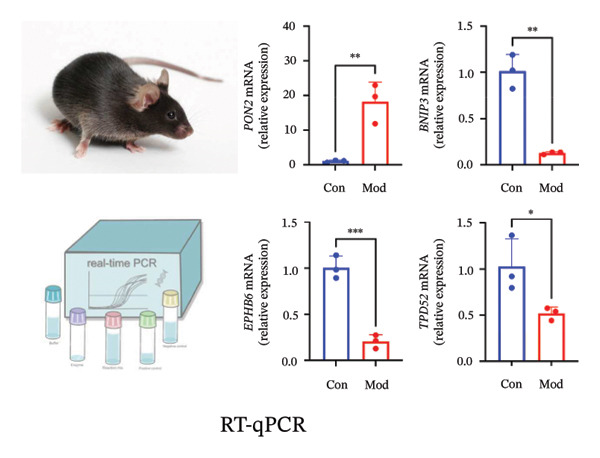
(g)

## 2. Materials and Methods

### 2.1. Data Acquisition

Using “AMD” and “Human” as keywords, we searched the GEO database and identified one microarray dataset (GSE29801) and one single‐cell RNA‐seq dataset (GSE188280) for subsequent analyses. The microarray dataset GSE29801 was generated on platform GPL4133 and comprises macular and extramacular retina and RPE/choroid samples from eyes with no reported ocular disease, possible preclinical or early AMD, geographic atrophy (advanced dry AMD), and neovascular AMD, including 175 RPE/choroid samples and 118 retinal samples. From this dataset, we selected 96 samples (27 normal and 69 AMD) for downstream analyses; all AMD phenotypes were combined into a single “AMD” group and compared with normal samples in order to identify PANoptosis‐related alterations that are common across the AMD spectrum. The GSE188280 dataset consists of 12 samples from various regions of normal and AMD eyes, including the macula, peripheral retina, and choroid, and comprises single‐cell RNA‐seq data from two early‐stage AMD patients and matched controls, representing non‐advanced dry AMD [11]. Additionally, PANoptosis‐related genes were retrieved from Genecards using keywords such as apoptosis, pyroptosis, and necroptosis. After removing duplicates, a total of 4285 related genes were identified (Supporting File 1).

### 2.2. Single‐Cell Transcriptomic Analysis

Single‐cell RNA‐seq data from the human AMD dataset GSE188280, which comprises macular and peripheral retina and choroid samples from three dry AMD donors and age‐matched controls [12], were processed using the Seurat R package (Version 4.4.1). Quality control was applied prior to downstream analyses, and low‐quality cells were excluded; only cells with 300–8000 detected genes, 2000–10,000 unique molecular identifiers (UMIs), and a mitochondrial gene expression fraction of ≤ 15% were retained. The corresponding QC metrics are provided in Supporting Figures 2A and 2B. The gene expression matrix was subsequently normalized and scaled and, where appropriate, integrated across samples to mitigate batch effects. Highly variable genes were then identified using the FindVariableFeatures function (*n* = 2000) for principal component analysis (PCA) (Supporting Figure 2C), and the number of principal components (PCs) carried forward was determined using the ElbowPlot criterion. Clustering analysis was then performed by constructing a k‐nearest‐neighbor graph with the “FindNeighbors” function and applying the “FindClusters” function with an optimized resolution value to generate cell clusters. Uniform manifold approximation and projection (UMAP) was used to visualize the clustering results. For downstream analyses, all AMD cells were combined into a single “AMD” group and compared with control cells within each relevant cell type.

### 2.3. Differential Expression Analysis

DEGs were identified using the Seurat R package’s “FindMarkers” function [13]. We analyzed gene expression heterogeneity between different cell types and between AMD and control samples. Statistical significance was determined by an adjusted *p*‐value < 0.05 and the default Seurat threshold of an average |log2 fold change| ≥ 0.5. Additionally, differential expression analysis was performed using the “Limma” package for the GSE29801 dataset, with selection criteria of |log2FC| > 1.0 and *p*‐value < 0.05. The DEGs were then visualized using the “ggplot2” package.

### 2.4. Cell Type Annotation and Gene Set Scoring

Based on cell‐specific markers derived from publicly available literature, the major cell populations were manually annotated. Using a PANoptosis‐related gene set, the annotated cell populations were scored with five single‐cell scoring methods: singscore, AddModuleScore, AUCell, ssGSEA, and UCell. The cell population with the highest score was re‐annotated, and t‐SNE analysis was performed for visualization.

### 2.5. Transcription Factor (TF) Identification

The GENIE3 package was employed to compute co‐expression networks from gene expression data, with the goal of inferring gene regulatory networks from high‐dimensional gene expression data [14]. The RcisTarget package was used to analyze potential binding motifs of TFs. RcisTarget integrates known TF binding site data with genomic sequences and transcriptomic information to predict the binding sites of TFs on target genes and their regulatory effects. Additionally, to assess the activity of regulatory factors in each cell, the AUCell package was applied to calculate the regulatory factor activity score for each cell. AUCell evaluates the relative level of specific TF activity in each single cell through gene set enrichment analysis, thereby providing regulatory information at the single‐cell level.

### 2.6. Cell Trajectory Analysis

In this study, the Monocle 3 package was used to perform “pseudotime” ordering of single cells [15], positioning the cells along an inferred developmental trajectory. The reverse graph embedding (RGE) method was applied to reduce the dimensionality of the data, successfully mapping the high‐dimensional gene expression data to a lower‐dimensional space and revealing the state transitions of cells during the developmental process. The pseudotime value of each cell reflects its progression along the inferred trajectory, providing a quantitative analysis of cell fate determination and transitions.

### 2.7. Cell–Cell Communication Analysis

The R package “CellChat” was employed to analyze the crosstalk between all cell types. It systematically examines the signaling pathways between cells, uncovering the interactions and regulatory mechanisms among different cell populations. The package utilizes single‐cell RNA sequencing data, integrating known ligand–receptor interaction networks, to perform a quantitative analysis of intercellular communication. CellChat offers various functionalities, such as calculating communication strength between cells, identifying key communication pathways, and predicting potential interaction patterns between different cell types.

### 2.8. MR Analysis Between Signature Genes and AMD

We utilized a two‐sample MR method, employing single nucleotide polymorphisms (SNPs) as instrumental variables (IVs), to explore the causal association between characteristic genes and the risk of AMD. The data were sourced from the OpenGWAS database of the Integrative Epidemiology Unit (IEU). We assessed the association between characteristic genes and the risk of AMD using the “TwoSampleMR” package and the inverse variance weighting (IVW) method. To ensure the reliability of our results, heterogeneity was tested using Cochran’s *Q* test, and gene pleiotropy was evaluated with the Pleiotropy Pval test, thereby confirming the robustness of our findings.

### 2.9. Machine Learning

For the PANoptosis‐related DEGs, we employed three machine learning algorithms: LASSO, SVM‐RFE, and RF, for gene selection. LASSO regression is a variant of linear regression that applies an L1 regularization term to the regression coefficients, enabling variable selection and controlling model complexity. LASSO performs well with multivariate data, effectively identifying the most predictive genes while avoiding overfitting. Its inherent sparsity ensures that the selected genes in the model provide high interpretability. Support vector machine (SVM) is a supervised learning method designed to find a hyperplane that maximizes the margin between different categories. SVM can handle high‐dimensional data and, through the kernel trick, efficiently maps nonlinear classification problems, extracting meaningful features from complex gene expression data [16]. Random forest (RF) is an ensemble learning method based on decision trees, constructing multiple decision trees and voting on their results to improve classification accuracy and reduce overfitting. RF is highly resistant to noise and performs well in classification tasks, particularly when handling gene selection problems with a large number of features and complex data structures, making it effective in identifying potential key genes.

### 2.10. Nomogram Model Construction

We constructed a nomogram model using the R package “rms,” with selected diagnostic genes as feature variables, and performed regression analysis to generate a nomogram for risk prediction. To assess the accuracy and reliability of the model, we validated its performance using calibration curves, examining the fit between predicted probabilities and actual observed values. Additionally, to quantify the net benefit of the model at different clinical decision thresholds, we conducted decision curve analysis (DCA) using the “decision_curve” function from the R package “rmda.” The DCA results provided a quantitative assessment of the net benefit for different intervention strategies, further validating the model’s feasibility and potential for clinical applications and offering scientific decision support for gene screening and disease prediction.

### 2.11. Shapley Additive Explanations

In this study, we employed shapley additive explanations (SHAP), an advanced model interpretability tool based on game theory, to quantify the contribution of each feature in the predictions of machine learning models. SHAP values accurately reflect the influence of each feature on the model’s decision‐making process by fairly distributing the “payoff” (i.e., the changes in the model’s output). By applying SHAP analysis, we can uncover the importance of core genes in disease risk prediction models, deepening our understanding of how these genes biologically influence disease progression and providing a more precise explanation of disease mechanisms and potential therapeutic targets.

### 2.12. Gene Set Enrichment Analysis (GSEA)

GSEA is a computational method used to determine whether a predefined gene set is statistically enriched in the top‐ or bottom‐ranked genes within a specific biological state. This method ranks expression data and evaluates the cumulative enrichment score (CES) of particular gene sets. A key feature of GSEA is its ability to effectively identify biological processes or functional pathways that are statistically correlated with a given phenotype or experimental condition, without relying on traditional differential expression analysis.

### 2.13. Gene Set Variation Analysis (GSVA)

GSVA is an unsupervised bioinformatics method used to estimate changes in gene set activity across individual samples. GSVA calculates the Enrichment Score (ES) of gene sets, providing a quantitative tool to assess gene set expression differences across various biological samples. A key advantage of this method is its nonparametric nature and its ability to operate without relying on predefined classifications, making it particularly effective for exploring disease‐related transcriptomic data.

### 2.14. Animals

Ten male C57BL/6 mice (SPF grade), aged 6–8 weeks and weighing 18–22 g, were purchased from Henan Skebes Biotechnology Co., Ltd. (License No. SCXK [Yu] 2020‐0005). All animals were housed in the Laboratory Animal Technology Centre of Jiangxi University of Traditional Chinese Medicine (Facility License No. SYXK [Gan] 2022‐0002). Animals were maintained under controlled conditions (temperature: 24°C–28°C; humidity: 60%–75%) with ad libitum access to food and water. Bedding was replaced twice per week.

### 2.15. Main Reagents and Instruments

Sodium iodate powder (NaIO_3_) was obtained from Sigma‐Aldrich (Shanghai); the BCA protein quantification kit and RIPA lysis buffer were purchased from Beyotime Biotechnology (Shanghai, China); protein molecular weight markers from Thermo Fisher Scientific; the reverse transcription kit from TaKaRa (Japan); primers for PON2, BNIP3, EPHB6, TPD52, and β‐actin were synthesized by Tsingke Biotechnology Co., Ltd.; the surgical microscope was provided by ZEISS (Germany); and the PCR thermal cycler was manufactured by Roche.

### 2.16. Grouping, Modeling, and Sampling

After one week of adaptive feeding, ten C57BL/6 mice were randomly divided into two groups using a random number table: a control group (*n* = 10) and a model group (*n* = 10). The control group received intraperitoneal injections of normal saline, while the model group was injected intraperitoneally with sodium iodate (NaIO_3_) at a dose of 30 mg/kg for 1 week to induce the AMD model. At the end of the experiment, the animals were euthanized, and the eyeballs were collected. The cornea, lens, vitreous body, optic nerve, and anterior segment were removed. A 2 × 4 mm section of the eye wall—including the retina, choroid, and sclera—was excised from both sides of the optic disc and stored at −80°C for further analysis.

### 2.17. RT‐PCR

Total RNA was extracted from the retinal tissues of ten randomly selected mice per group using an animal tissue RNA extraction kit, following the manufacturer’s protocol. After determining RNA concentration, cDNA was synthesized using a reverse transcription kit. The relative mRNA expression levels of PON2, BNIP3, EPHB6, and TPD52 were quantified using the 2^−ΔΔCt^ method, with β‐actin serving as the internal reference gene. The upstream primer for β‐actin was 5′‐GTG​ACG​TTG​ACA​TCC​GTA​AAG​A‐3′, and the downstream primer was 5′‐GTA​ACA​GTC​CGC​CTA​GAA​GCA​C‐3′. The upstream primer for PON2 was 5′‐AGG​AAT​CGA​AAC​TGG​AGC​TGA​G‐3′, and the downstream primer was 5′‐AGA​TCA​AAG​GCC​CCA​GCT​GAC‐3′. The upstream primer for BNIP3 was 5′‐TTC​CAG​CCT​CGC​TTC​TTA​TTA‐3′, and the downstream primer was 5′‐AAT​CTT​CCT​CAG​ACA​GAG​TGC​TG‐3′. The upstream primer for EPHB6 was 5′‐TCT​CAC​TCA​GCG​TGG​CTT​CTA​TG‐3′, and the downstream primer was 5′‐TGT​CTC​AGG​AAA​AGG​CAA​AGG‐3′. The upstream primer for TPD52 was 5′‐AGG​AAG​GAG​AGG​ATG​CTG​TTA​CC‐3′, and the downstream primer was 5′‐CTT​CTC​TTT​TGC​GGC​CAA​TAC​TT‐3′.

### 2.18. Statistical Methods

Statistical analysis was performed using GraphPad Prism 9 software. The data were normally distributed with homogeneity of variance, and *t*‐tests were applied. A *p*‐value of < 0.05 was considered statistically significant, while a *p*‐value of < 0.01 was considered highly significant.

## 3. Results

### 3.1. Dimensionality Reduction for Annotating Cell Types

To gain a deeper understanding of the cellular heterogeneity, diversity, and transcriptomic alterations between patients with AMD and healthy controls, we performed a comprehensive analysis of single‐cell transcriptomic data from retinal regions. Cells were isolated from the macula and peripheral regions and subsequently subjected to scRNA‐seq. During preliminary data processing, we first performed quality control (Supporting Figure 2A and 2B). Next, 2000 highly variable genes were selected using the “FindVariableFeatures” function for subsequent analysis (Supporting Figure 2C). We then used 15 PCs to cluster cells with similar gene expression profiles, ultimately identifying 15 distinct cell populations. The top five significant genes for each population were visualized using heatmaps (Figure 2(a)). For further cell type identification, we performed detailed manual annotation and dimensionality reduction using the UMAP algorithm (Supporting File 3), identifying six major cell types: endothelial cells (EC), RPE cells, Schwann cells, mast cells, macrophages (Mac), and fibroblasts (Fibro) (Figure 2(b)). We additionally provide a UMAP visualization including all cells, colored by group (AMD vs. control) and by sample, in Supporting Figure 4A–4C. The distribution of each cell type across different samples was displayed in bar charts, highlighting the differences in cellular heterogeneity between AMD patients and healthy controls (Figure 2(c)).

FIGURE 2Dimensionality reduction annotation of GSE188280. (a) Clustering heatmap. (b) U‐map dimensionality reduction annotation plot of 6 cell groups. (c) Stacked bar plot of the proportion of 6 cell groups in each sample. (d) Volcano plot of differentially expressed genes. (e) Single‐cell scoring violin plot for the control group. (f) Single‐cell scoring violin plot for the AMD group. (g) Pseudotime analysis plot. (h) Single‐cell scoring U‐map plot. (i) T‐SNE plot of macrophage secondary annotation.

**Figure:**
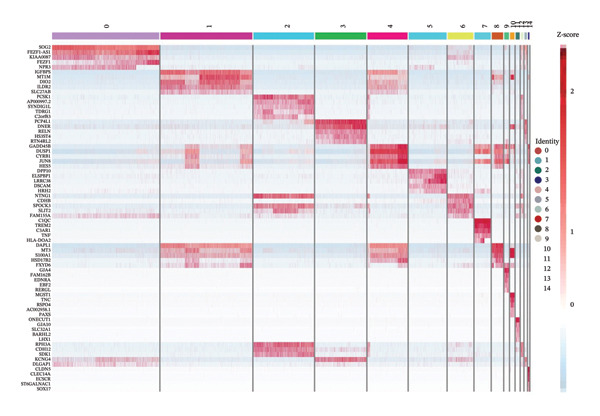
(a)

**Figure:**
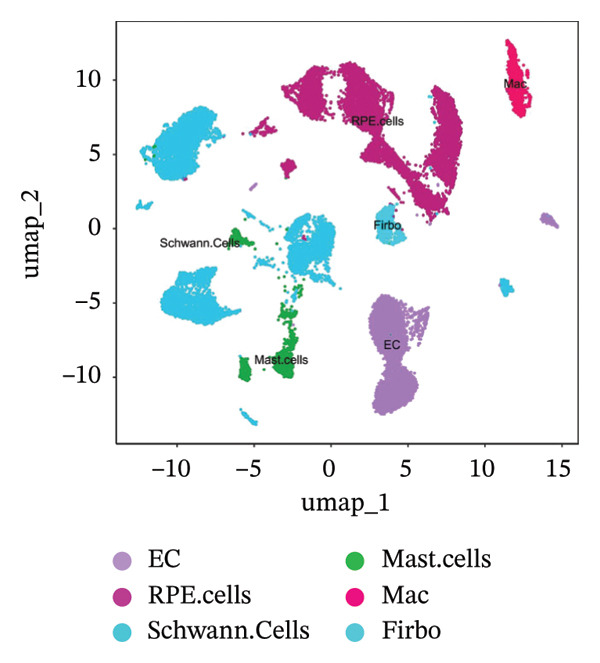
(b)

**Figure:**
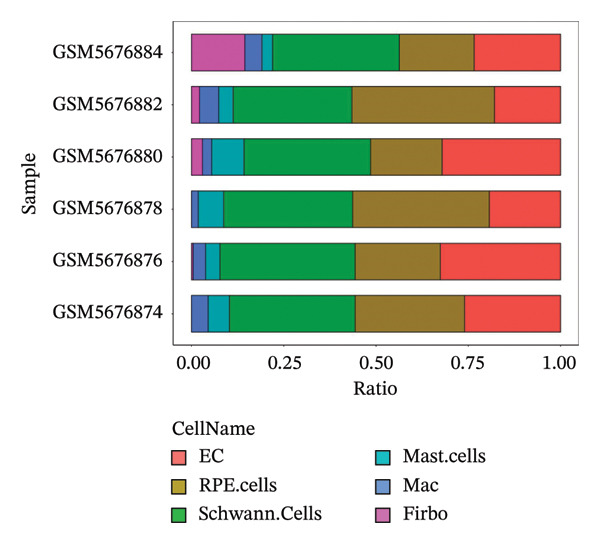
(c)

**Figure:**
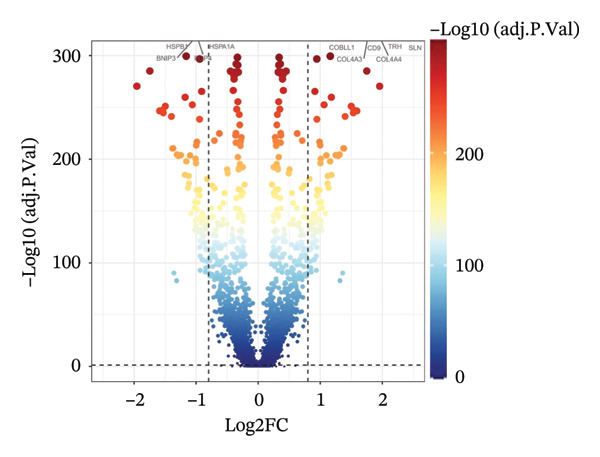
(d)

**Figure:**
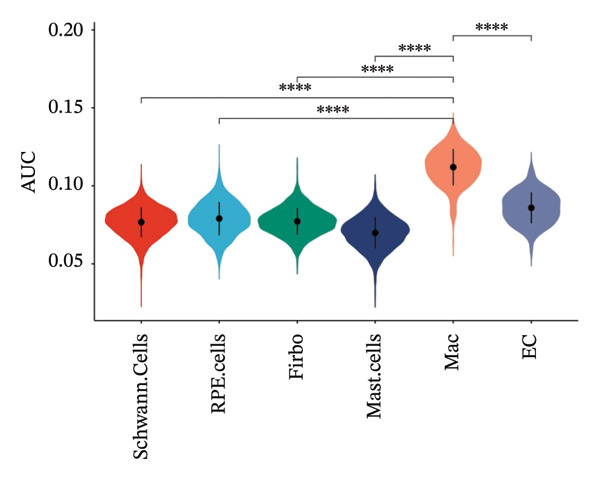
(e)

**Figure:**
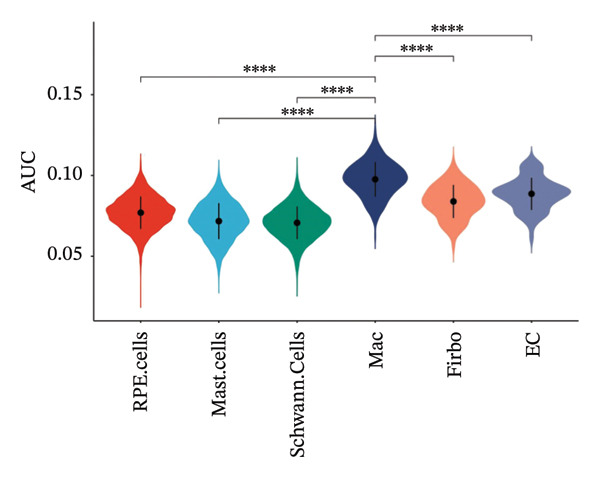
(f)

**Figure:**
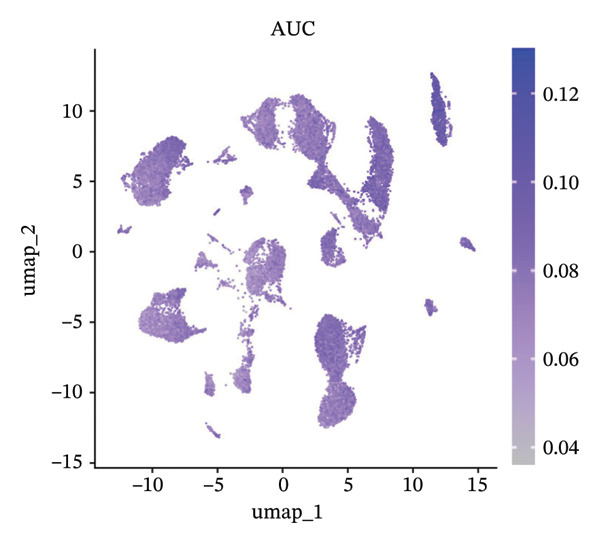
(g)

**Figure:**
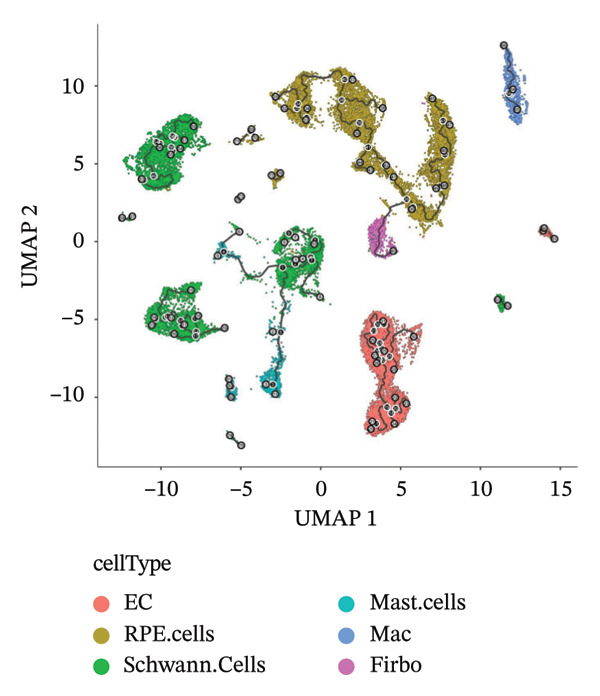
(h)

**Figure:**
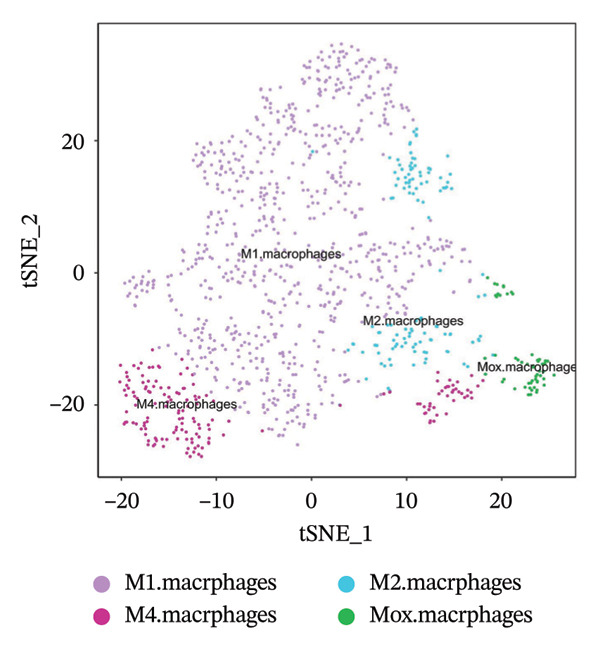
(i)

Furthermore, we used the “FindMarkers” function from the R package “Seurat” to identify 232 DEGs between the AMD and control groups, which were visualized through a volcano plot (Figure 2(d)). Pseudotime analysis further revealed potential transformation relationships between cell types (Figure 2(h)). Given the roles of mast cells and macrophages in immune responses and tissue repair, this transformation may reflect adaptive changes in cellular function.

### 3.2. Gene Set Scoring for Each Cell Cluster and Secondary Annotation

In this study, we utilized five single‐cell scoring methods (singscore, AddModuleScore, AUCell, ssGSEA, and UCell) to assess cell populations annotated based on a pan‐apoptosis‐related gene set. The results consistently showed that macrophages had the highest scores across all cell populations, suggesting their potential key role in the pan‐apoptotic process during AMD disease progression (Figures 2(e), 2(f), 2(g)). Subsequently, the macrophage population was extracted and manually re‐annotated into subpopulations, including M1, M2, M4, and Mox. t‐SNE visualization was then performed to analyze the data (Figure 2(i)). Notably, M1‐type macrophages accounted for the majority of the macrophage subpopulations, indicating their potentially dominant role in the pan‐apoptotic process during AMD progression.

### 3.3. Transcription Factor Regulatory Network

To further investigate the potential molecular mechanisms in AMD, we performed SCENIC analysis. The heatmap of regulatory network activity across cell types highlights differences in regulatory network activity between various cell types. Among them, MLX and TCF3_extended show higher activity in specific cell types, such as macrophages, while ZBTB7A exhibits strong activity in fibroblasts. This heatmap reveals cell‐type‐specific regulatory networks, indicating that certain regulatory networks are more active in specific cell types, reflecting the complexity of gene regulation in different cellular contexts (Figures 3(a) and 3(b)). The heatmap of AUC scores between regulatory networks and cell types displays the AUC values across various regulatory networks and random cell subsets. AUC serves as a performance metric for evaluating the ability of regulatory network activity to predict cell type classification. Regulatory networks such as GABPA, SMAD1_extended, and ZBTB25_extended exhibit higher AUC values, suggesting that their regulatory activity is more predictive of cell type classification. The lower AUC values for other regulatory networks indicate weaker predictive power for cell types, likely due to low or more widespread expression patterns of these networks (Figure 3(c)).

FIGURE 3Transcription factor regulatory network and cell‐cell communication in single‐cell analysis. (a–c) Transcription factor prediction heatmaps. (d) Interaction net count plot of AMD cells. (e) Interaction net weight plot of AMD cells. (f) Detailed network of cell–cell interactions among 6 cell subsets. (g) Chord diagram and heatmaps of the TGFβ signaling pathway across 6 cell groups. (h) Heat map showing the inferred signaling roles of each cell group in the TGFβ signaling pathway network. (i) Heat map of TGFβ pathway cell–cell communication, displaying the inferred communication strength between source and target across the six cell groups.

**Figure:**
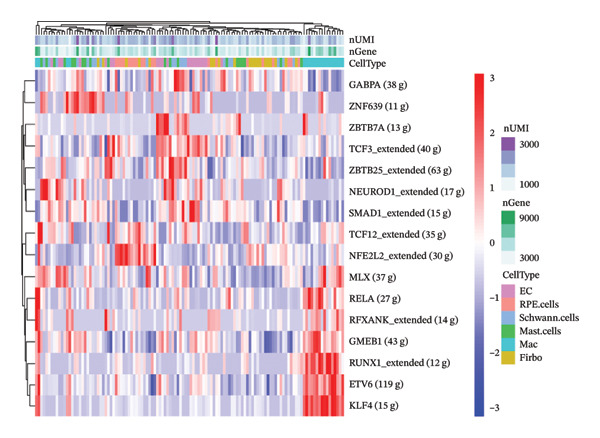
(a)

**Figure:**
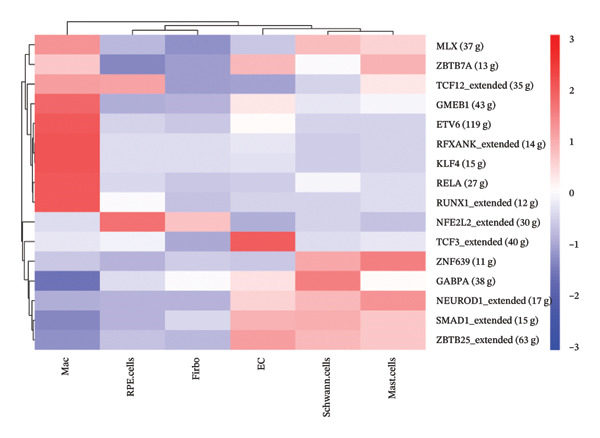
(b)

**Figure:**
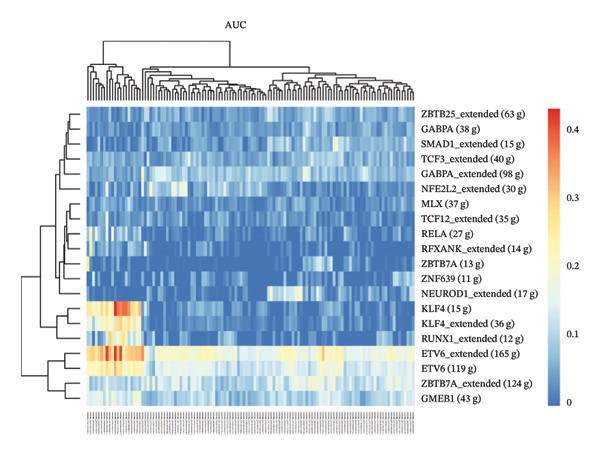
(c)

**Figure:**
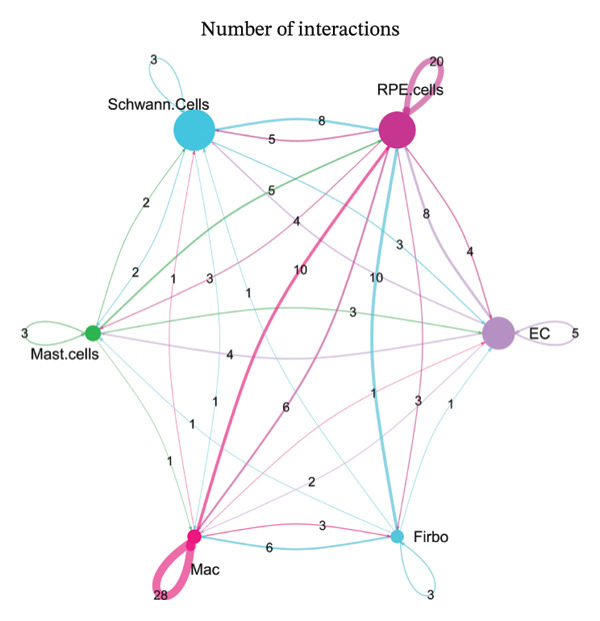
(d)

**Figure:**
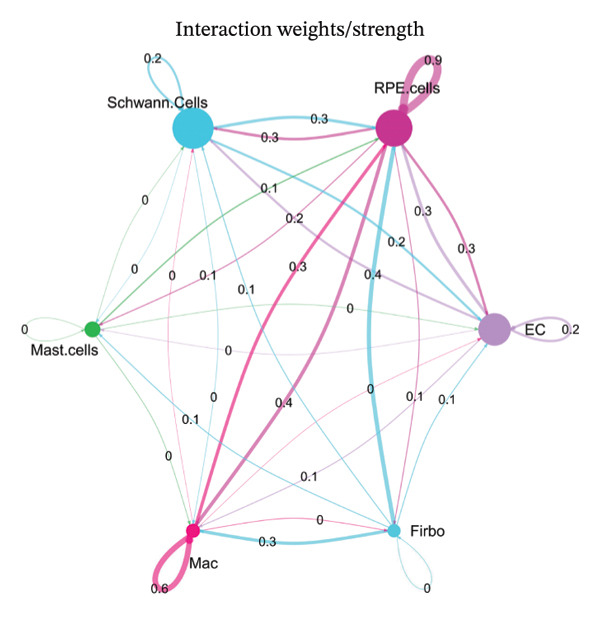
(e)

**Figure:**
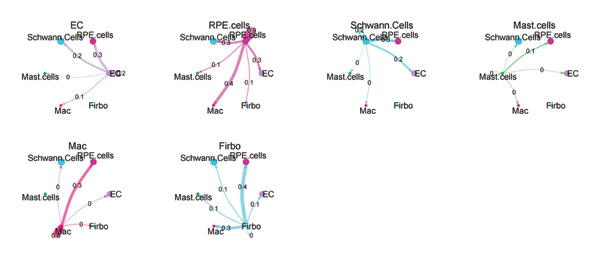
(f)

**Figure:**
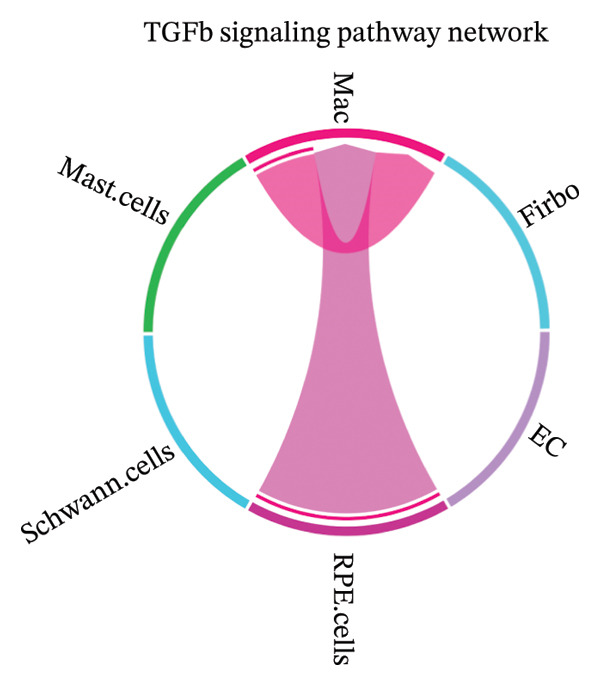
(g)

**Figure:**
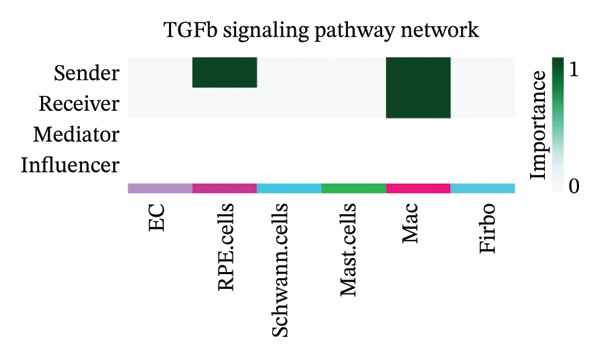
(h)

**Figure:**
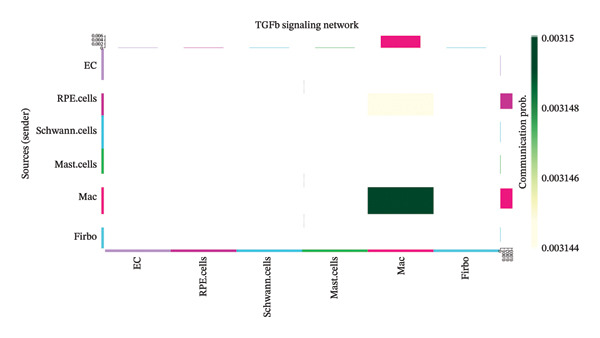
(i)

### 3.4. Cell‐to‐Cell Communications

To gain a deeper understanding of how cells interact and contribute to the pathogenesis of AMD in patients, we investigated cell communication through ligand–receptor interactions. In the cell communication network map based on the number of connections, Mac plays a central role, interacting most frequently with other cell types, particularly RPE cells, with 28 interactions. RPE cells exhibit strong communication with several cell types (such as Mac, Schwann cells, ECs, and others), highlighting their significance within this network (Figure 3(d)). In the cell communication network map based on connection strength, RPE cells demonstrate the highest communication strength with themselves, reaching 0.9, indicating very strong interactions within the RPE cell population. Additionally, RPE cells show robust communication links with multiple other cell types, particularly with Mac and fibroblasts, where the interaction strength is 0.4 (Figures 3(e) and 3(f)).

Subsequently, we explored the interactions, communication probabilities, and roles of different cell populations in the TGFβ signaling pathway. Figure 3(g) presents a chord diagram illustrating the signal transmission relationships between cell populations in the TGFβ signaling network. The diagram shows significant differences in signal transmission between EC, RPE cells, Schwann cells, mast cells, Mac, and Fibro, with some cell populations exhibiting stronger interactions. Notably, there is a close linkage between Mac and Fibro, while signal transmission between other cell populations is relatively weaker. This result provides important visual evidence for understanding the complexity of TGFβ signaling in cell communication. Figure 3(h) displays the communication probabilities between cell populations in the TGFβ signaling pathway. Heatmap analysis visually reveals the communication strength between different cell populations as “senders” and “receivers.” In particular, the signal transmission probability between Mac and Fibro is significantly higher than between other cell populations, suggesting a more intimate exchange between these two cell types in the signaling network. Meanwhile, communication probabilities between other cell populations are lower, indicating their relatively weaker role in the TGFβ signaling pathway. This quantitative communication probability analysis offers systematic numerical support for the observed differences in signal transmission strength between cell types. Figure 3(i) illustrates the roles and importance of different cell populations in the TGFβ signaling pathway. The color gradient represents the contribution of each cell population to the signaling network, with dark green indicating higher importance and light green indicating lower importance. From the figure, it is evident that RPE and EC cells play crucial roles in the TGFβ signaling network, with high network importance, while other cells have relatively minor roles. This analysis provides deeper insights into the contributions of each cell population in TGFβ signal transduction, further highlighting their key roles in cell communication.

### 3.5. Identification and Enrichment Analysis of PANoptosis‐Related DEGs

Differential analysis of GSE29801 revealed 2391 DEGs, which were visualized using a volcano plot (Figure 4(a)). Subsequently, the intersection between the DEGs obtained from single‐cell analysis and PANoptosis‐related genes was determined, and a Venn diagram was created, identifying 29 apoptosis‐related DEGs (Figure 4(b)). These 29 genes were then subjected to GO and KEGG enrichment analysis. The results showed significant enrichment in several GO terms, including NADH regeneration, canonical glycolysis, postsynaptic density, asymmetric synapse, and cadherin binding. KEGG analysis revealed significant enrichment in pathways such as glycolysis and gluconeogenesis, biosynthesis of amino acids, and carbon metabolism (Figures 4(c) and 4(d)).

FIGURE 4Differential analysis and enrichment analysis. (a) Volcano plot of differentially expressed genes in dataset GSE29801. (b) Venn diagram of differentially expressed genes and PANoptosis‐related genes. (c) GO enrichment analysis circle plot. (d) KEGG enrichment analysis bubble plot.

**Figure:**
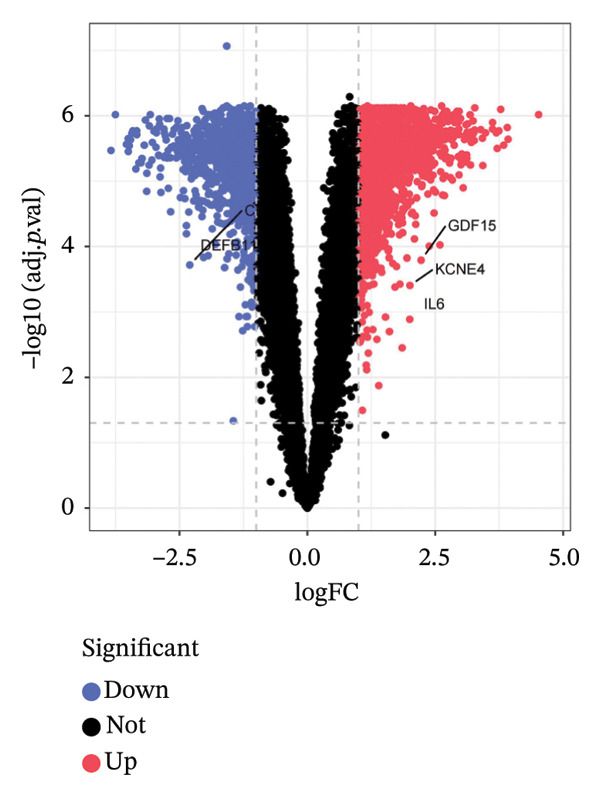
(a)

**Figure:**
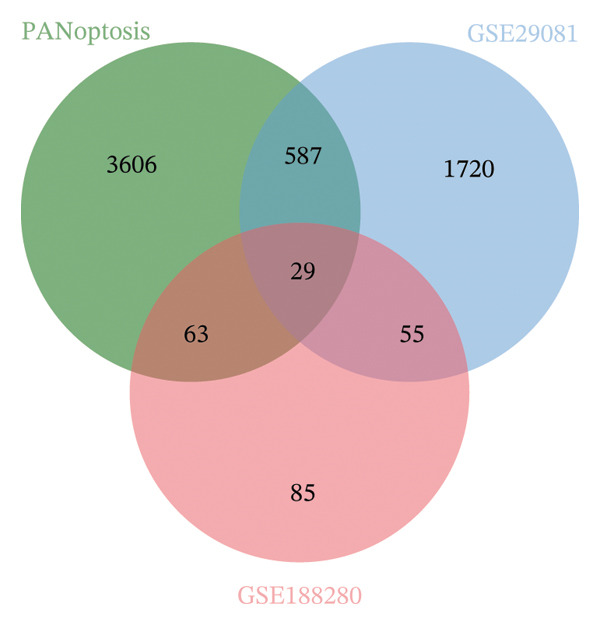
(b)

**Figure:**
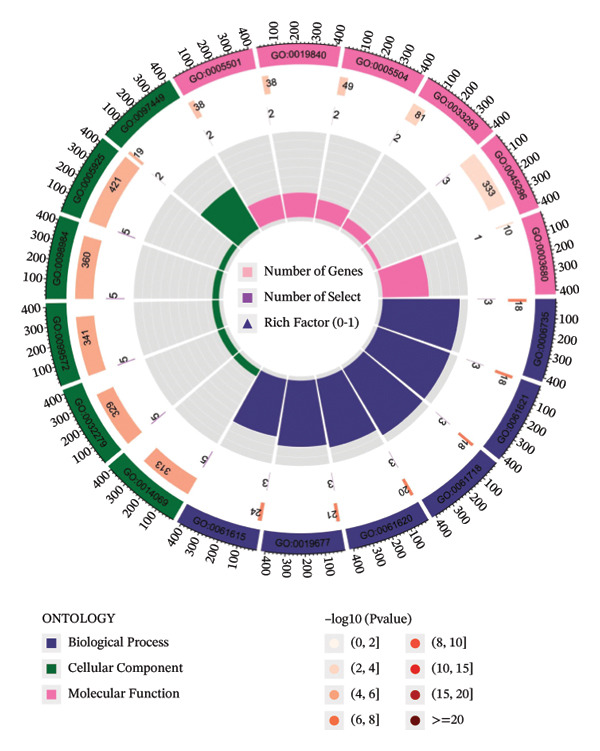
(c)

**Figure:**
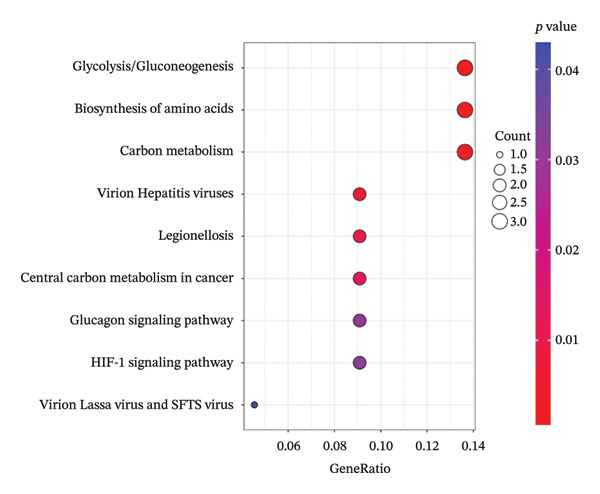
(d)

### 3.6. Mendelian Randomization and Colocalization Analysis

We identified the eQTLs regulating apoptosis‐related DEGs and used them as exposure factors for AMD outcomes. We performed a batch matching process and selected 86 SNPs that satisfied both the correlation and independence hypotheses as IVs for MR analysis. A positive causal relationship between TPD52 and AMD was identified (Figures 5(a), 5(b), 5(c), 5(d), 5(e)). TPD52 [IVW, OR = 1.158, 95% CI (1.030, 1.302), *p* = 0.014], and Bayesian colocalization analysis yielded PP.H1.abf = 0.976 (Figure 5(f)).

FIGURE 5MR analysis of PANoptosis‐related differentially expressed genes. (a) MR results for common genes significantly associated with AMD. (b) The forest plot of the causal effects of each SNP in TPD52 on the risk of AMD. (c) Funnel plot of TPD52 on AMD. (d) Leave‐one‐out plot of TPD52 on AMD risk when leaving one SNP out. (e) Scatter plot of the causal effect of TPD52 on the risk of AMD. (f) Bayesian colocalization of TPD52 and AMD.

**Figure:**
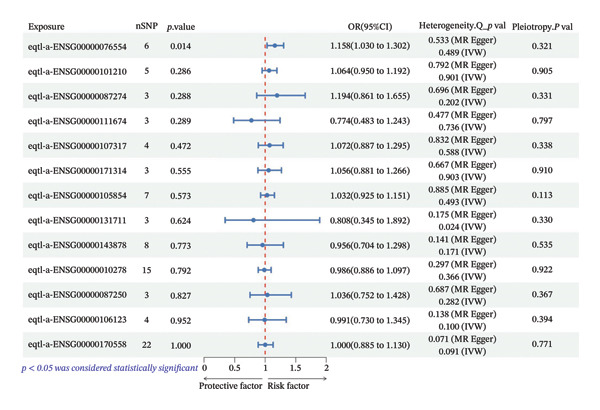
(a)

**Figure:**
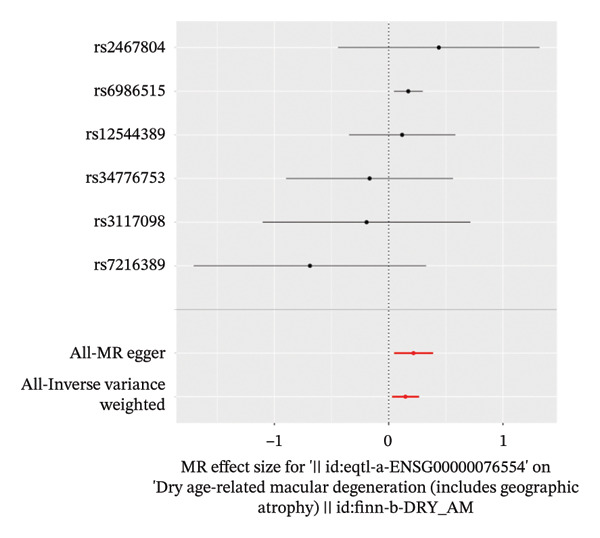
(b)

**Figure:**
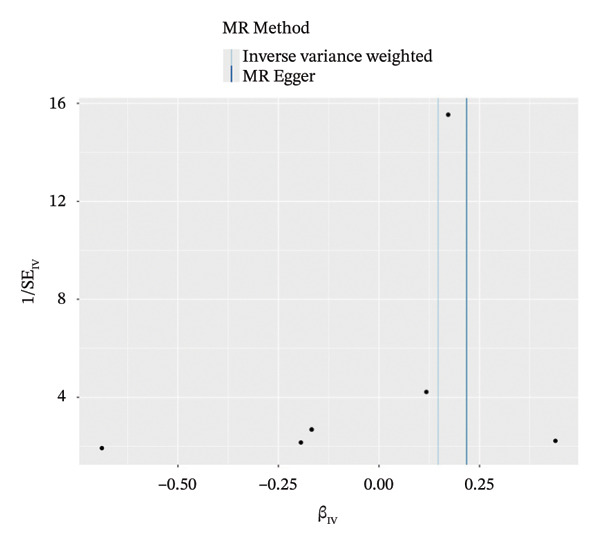
(c)

**Figure:**
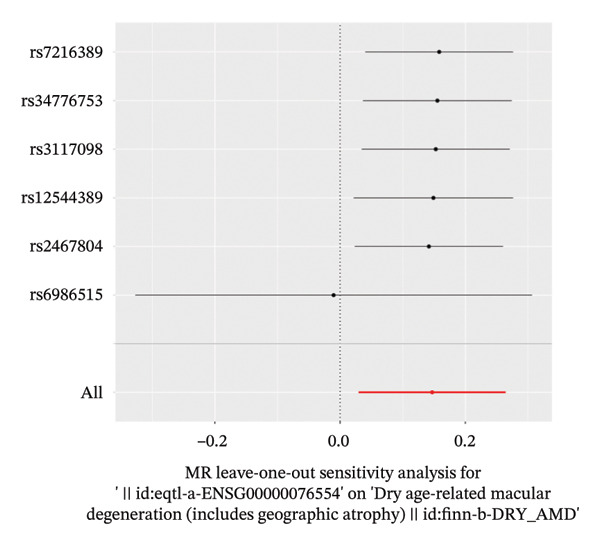
(d)

**Figure:**
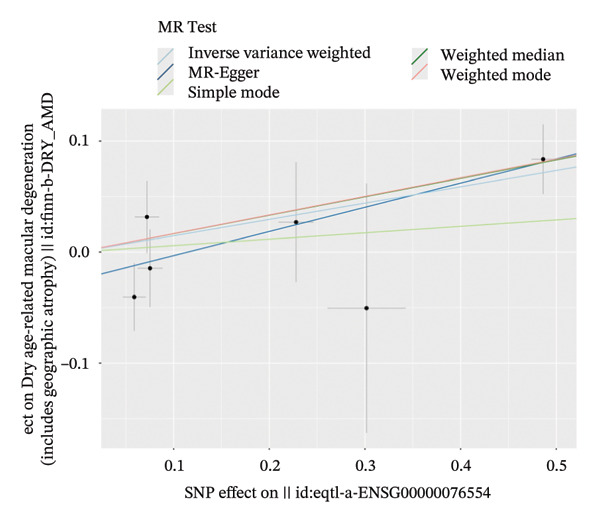
(e)

**Figure:**
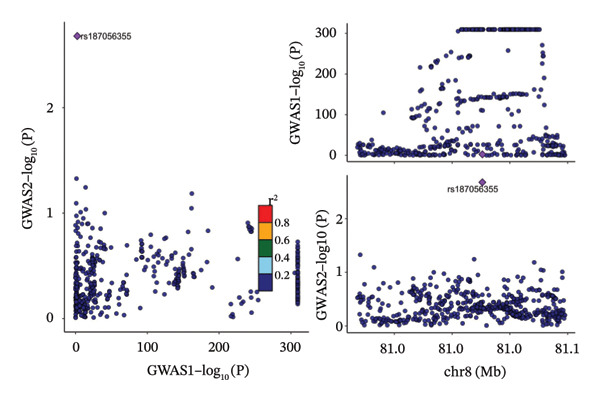
(f)

### 3.7. Acquisition of Core Genes and Development of Diagnostic Models

We employed three machine learning algorithms—LASSO, SVM‐RFE, and RF—to further screen the 29 apoptosis‐related DEGs. Using the SVM‐RFE method, we performed 10‐fold cross‐validation and identified 5 core genes. LASSO identified 6 core genes, while RF identified 7 targets (Figures 6(a), 6(b), 6(c), 6(d), 6(e)). After generating a Venn diagram, we selected genes that appeared in at least two algorithm‐based screenings as core genes, resulting in the identification of PON2, BNIP3, and EPHB6 (Figure 6(f)). Combining these core genes with those identified through Mendelian analysis, we established four core genes: PON2, BNIP3, EPHB6, and TPD52. Box plots demonstrated the expression level differences of these genes between the AMD and control groups in the GSE29801 dataset (Figure 7(a)), while a correlation matrix illustrated the expression level correlations among the four genes (Figure 7(b)). Furthermore, we constructed a nomogram based on these four core genes to predict AMD disease risk. Each gene’s expression level was assigned a different risk score, and a higher cumulative score predicted a greater disease risk (Figure 7(c)). Calibration curves showed that the model exhibited good predictive ability (Figure 7(d)). Additionally, DCA (Figure 7(e)) further validated the model’s net benefit across different threshold probabilities, demonstrating that it could effectively improve clinical decision‐making for disease risk prediction.

FIGURE 6Identify PANoptosis‐related hub genes from the shared candidate genes using machine‐learning algorithms. (a) LASSO coefficient trajectories. (b) Tenfold cross‐validation for LASSO to select the optimal *λ*. (c) SVM feature selection performance (10× CV accuracy versus number of features). (d) Random forest (RF) out‐of‐bag error versus number of trees. (e) RF variable‐importance ranking of candidate genes. (f) Venn diagram showing the overlap of genes identified by LASSO, SVM, and RF.

**Figure:**
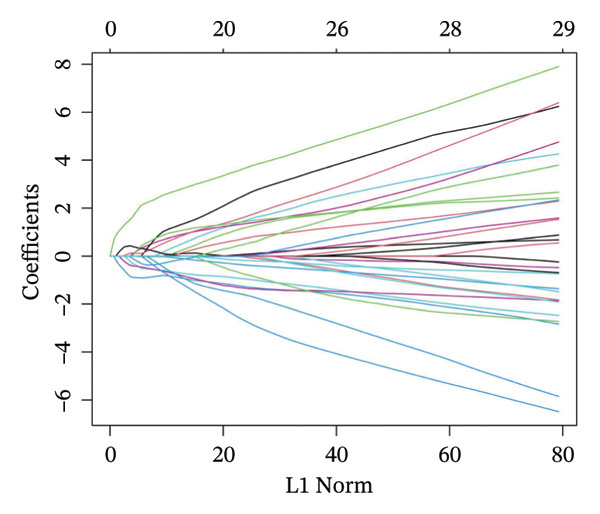
(a)

**Figure:**
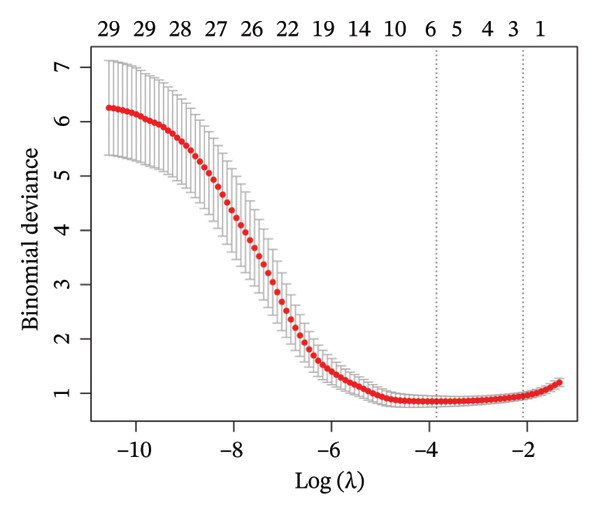
(b)

**Figure:**
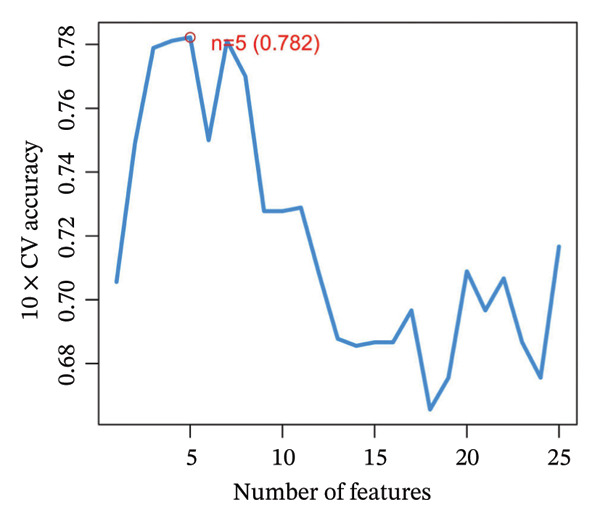
(c)

**Figure:**
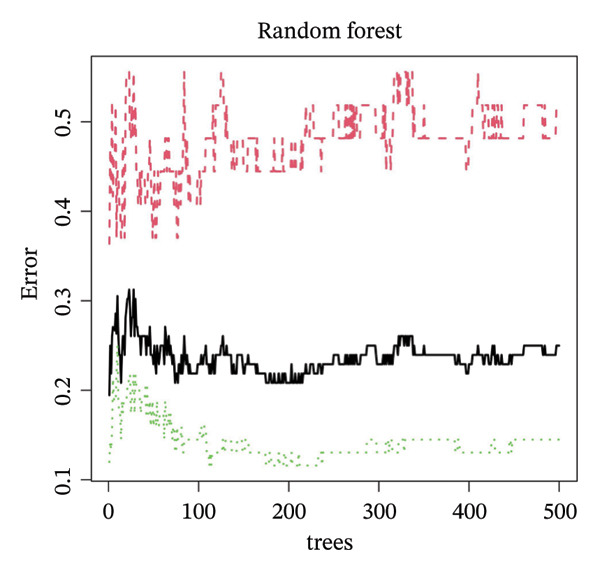
(d)

**Figure:**
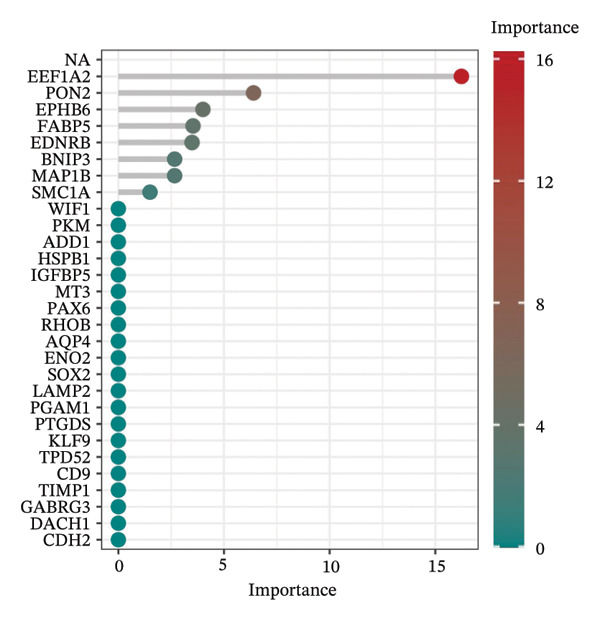
(e)

**Figure:**
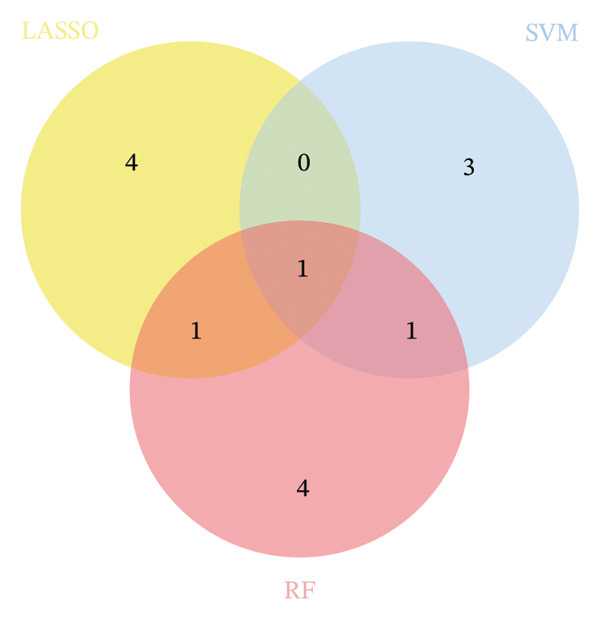
(f)

FIGURE 7Nomo diagnostic model of 4 hub genes (PON2, BNIP3, EPHB6, and TPD52). (a) Box plot of the expression levels of 4 hub genes. (b) Correlation plot of 4 hub genes. (c) The nomogram for predicting the risk of AMD. (d) Corrected calibration curve of nomogram for comparing predicted and actual probability of AMD. (e) DCA for the validation set.

**Figure:**
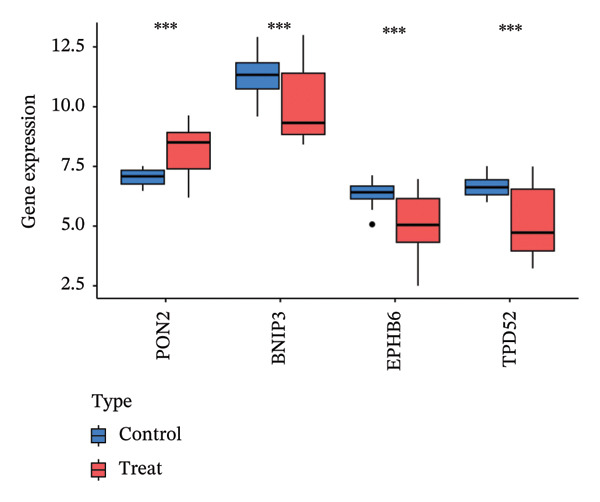
(a)

**Figure:**
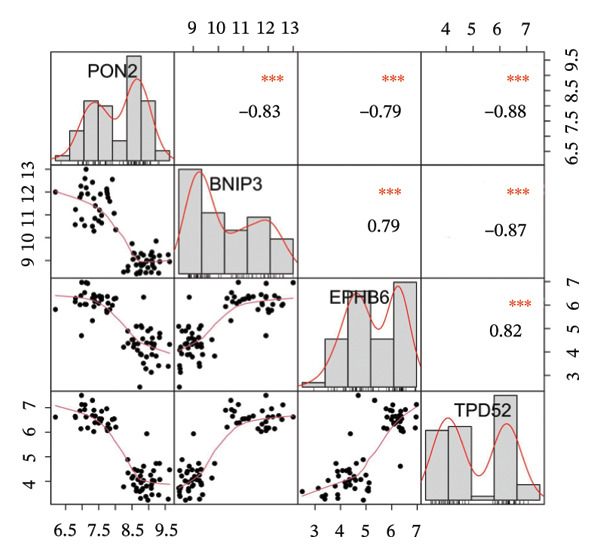
(b)

**Figure:**
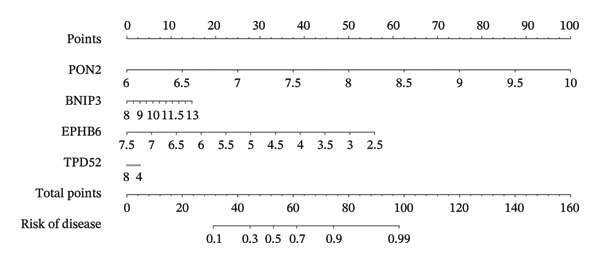
(c)

**Figure:**
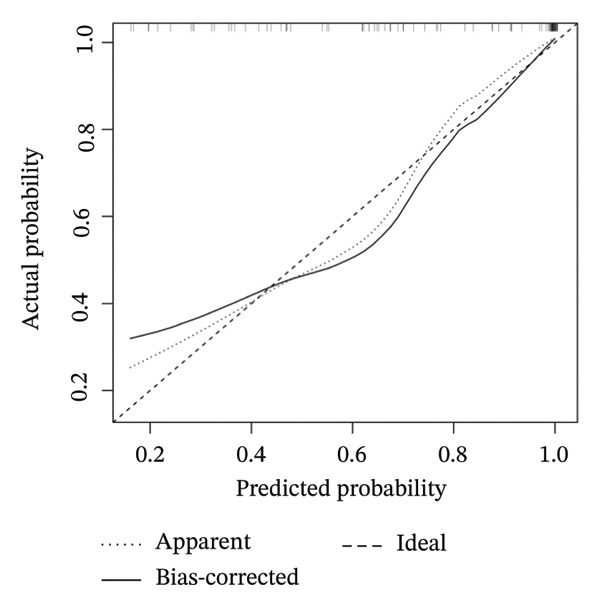
(d)

**Figure:**
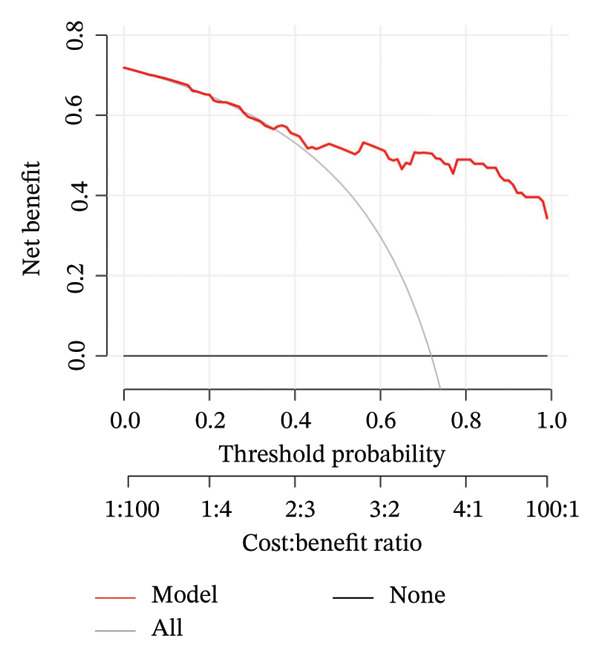
(e)

We divided the samples in the GSE29801 dataset into a training set (70%) and a validation set (30%) and trained and evaluated 10 different machine learning models. The evaluation results showed that the gradient boosting machine (GBM) model achieved the highest AUC value (0.938) (Figure 8(a)), indicating its superior performance in the classification task. In the feature importance analysis, SHAP bee and bar plots (Figures 8(b) and 8(c)) revealed the contribution of the four core genes to the model’s prediction, with PON2 having the largest impact among all genes, suggesting its crucial role in the prediction. Furthermore, the SHAP dependency plot (Figure 8(d)) revealed the interactions between genes. The strong interactions between PON2, EPHB6, and BNIP3 suggest that these genes may have mutually enhancing or inhibiting effects in the prediction. Finally, the SHAP force plot (Figure 8(e)) illustrated the negative contribution of each gene to the prediction for a single sample. The negative shifts of PON2, BNIP3, EPHB6, and TPD52 together caused the prediction for this sample to be lower than the baseline. This analysis provides an in‐depth understanding of the prediction mechanism of the model, clarifying the specific influence of each gene on the prediction results for individual samples.

FIGURE 8Shapley additive explanations plots. (a) ROC curves of 10 ML models. (b) Beeswarm plot of four hub genes based on SHAP analysis. (c) Bar plot of four hub genes based on SHAP analysis (d) SHAP dependency plots among hub genes. (e) SHAP waterfall plot.

**Figure:**
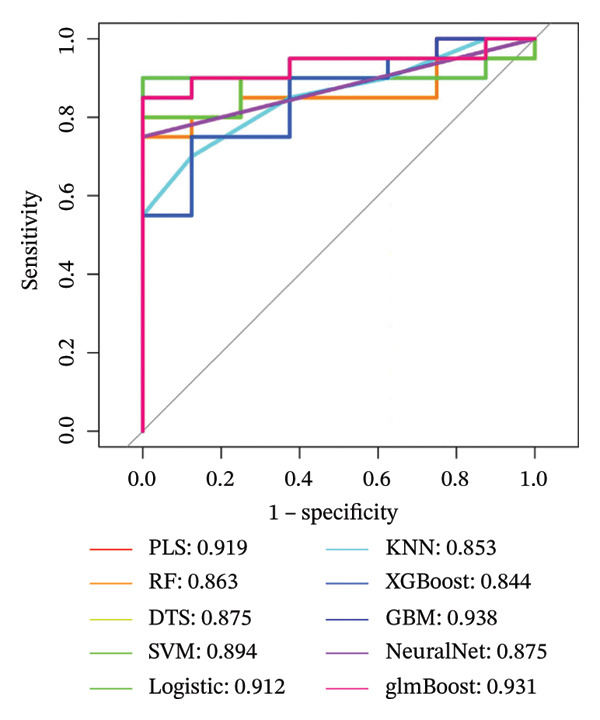
(a)

**Figure:**
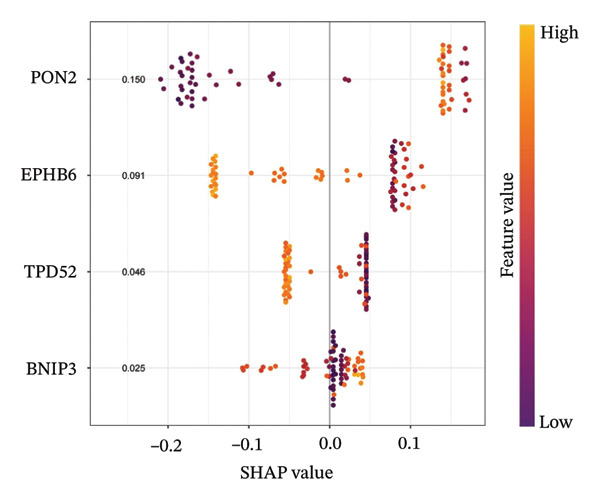
(b)

**Figure:**
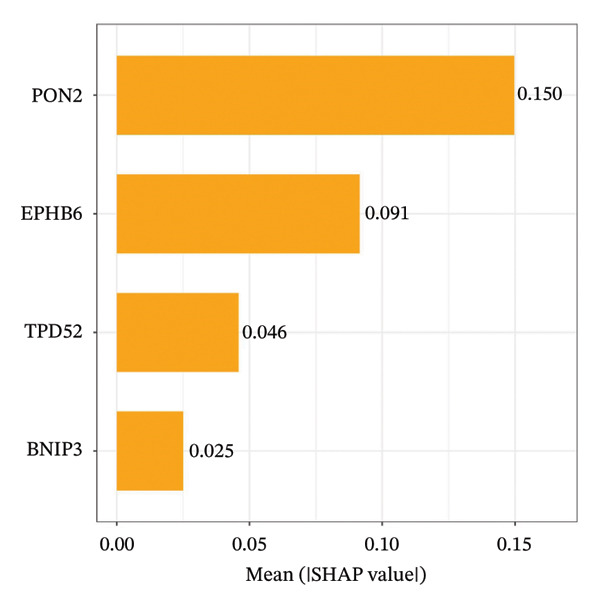
(c)

**Figure:**
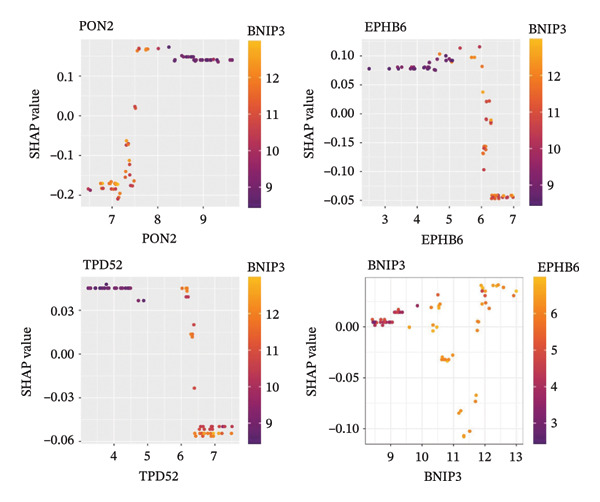
(d)

**Figure:**
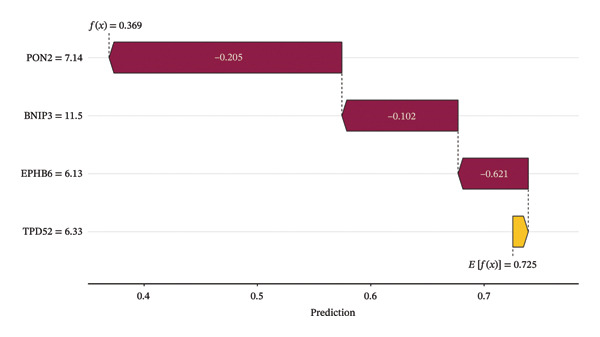
(e)

### 3.8. Results of GSEA and GSVA for Core Genes

We examined the signaling pathways enriched by the four hub genes to investigate their potential molecular mechanisms underlying the progression of AMD. GSEA analysis showed that PON2 was enriched in the complement and coagulation cascades, as well as the cytokine–cytokine receptor interaction signaling pathways (Figure 9(a)); BNIP3 was enriched in circadian rhythm, glycolysis, and gluconeogenesis signaling pathways (Figure 9(b)); EPHB6 was enriched in oxidative phosphorylation and glycosaminoglycan biosynthesis pathways (Figure 9(c)); and TPD52 was enriched in circadian rhythm, oxidative phosphorylation, and purine metabolism pathways (Figure 9(d)). Additionally, GSVA results showed that high expression of PON2 primarily activated ether lipid metabolism, extracellular matrix–receptor interaction, and TGF‐β signaling pathways (Figure 9(e)); high expression of BNIP3 was mainly enriched in oxidative phosphorylation, glycosaminoglycan biosynthesis, and alanine, aspartate, and glutamate metabolism signaling pathways (Figure 9(f)). Meanwhile, the expression of EPHB6 and TPD52 was significantly enriched in glycosaminoglycan biosynthesis, cardiac muscle contraction, and circadian rhythm pathways (Figures 9(g) and 9(h)). These results reveal that the hub genes may play a significant role in the pathological progression of AMD by regulating key signaling pathways associated with inflammation, metabolism, apoptosis, and extracellular matrix remodeling.

FIGURE 9GSEA and GSVA of hub genes. (a–d) GSEA analysis of hub genes (PON2, BNIP3, EPHB6, and TPD52). (e–h) GSVA analysis of hub genes (PON2, BNIP3, EPHB6, and TPD52).

**Figure:**
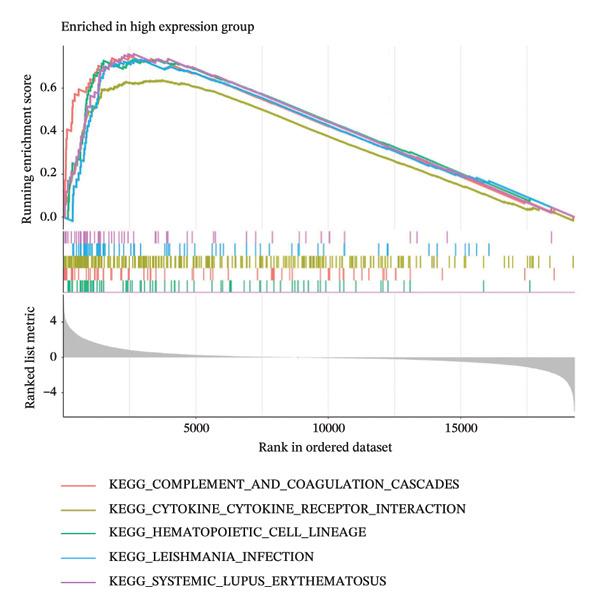
(a)

**Figure:**
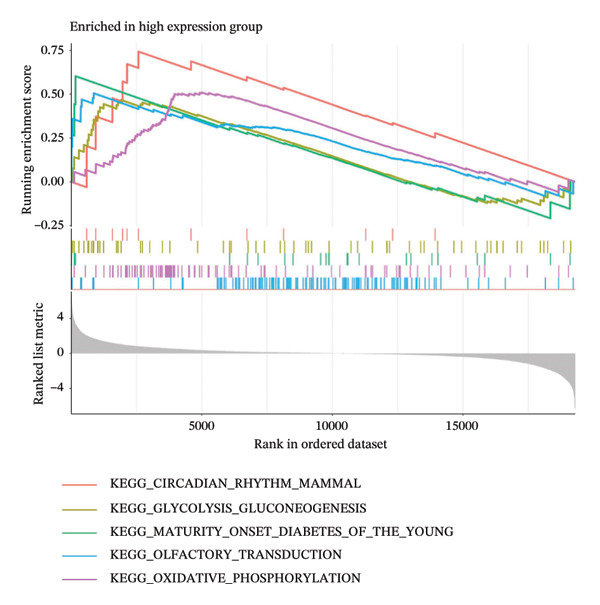
(b)

**Figure:**
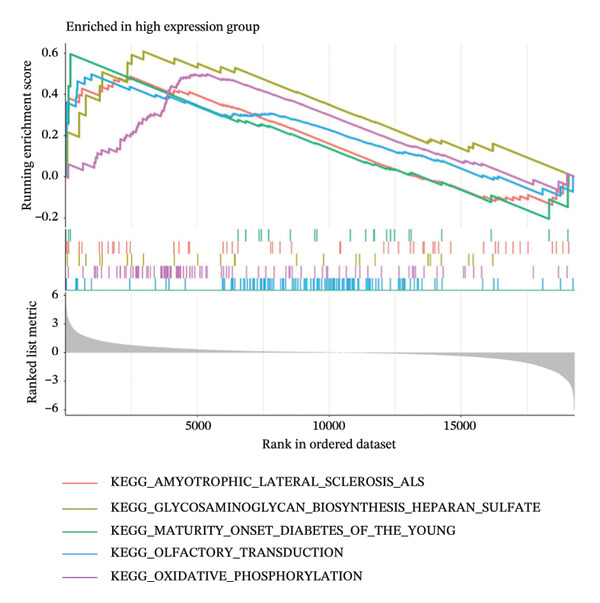
(c)

**Figure:**
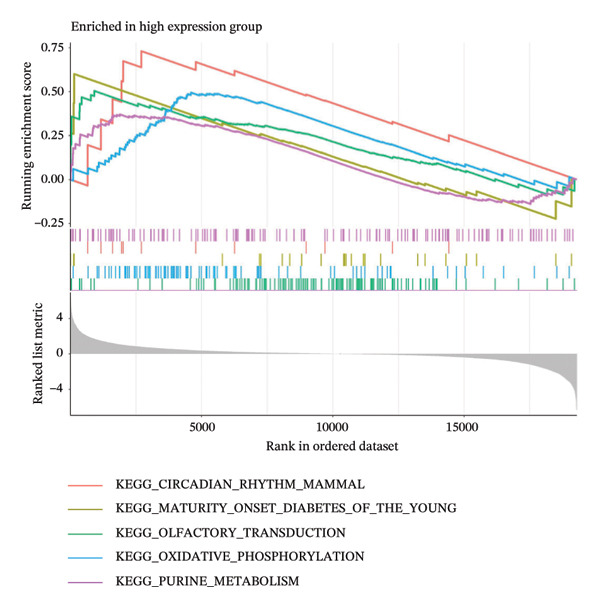
(d)

**Figure:**
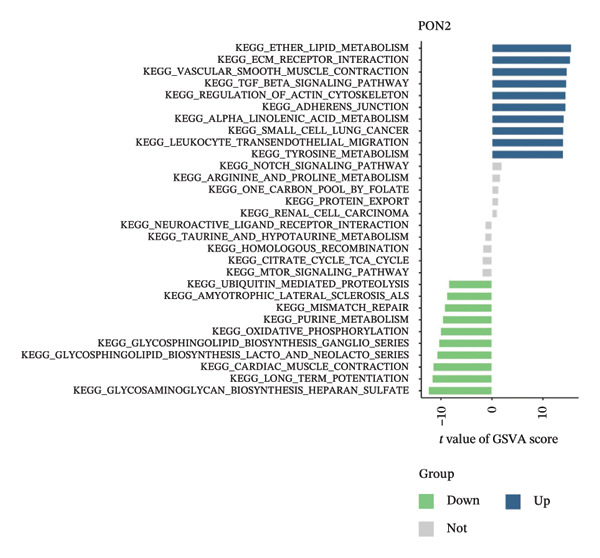
(e)

**Figure:**
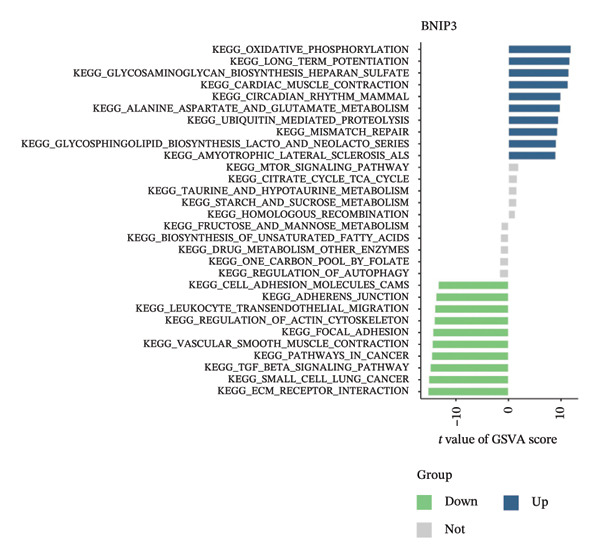
(f)

**Figure:**
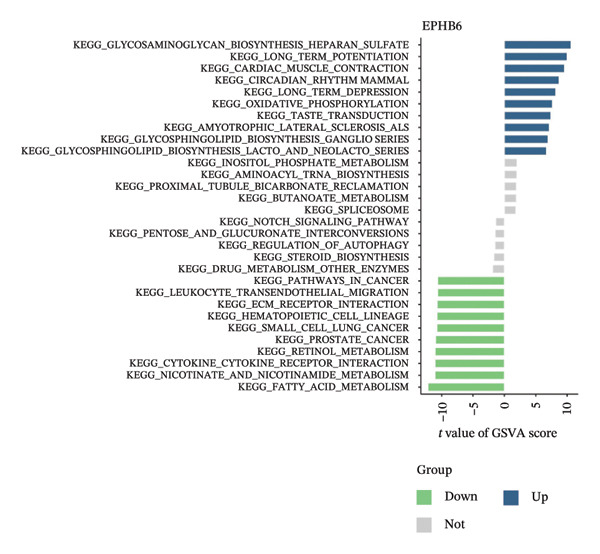
(g)

**Figure:**
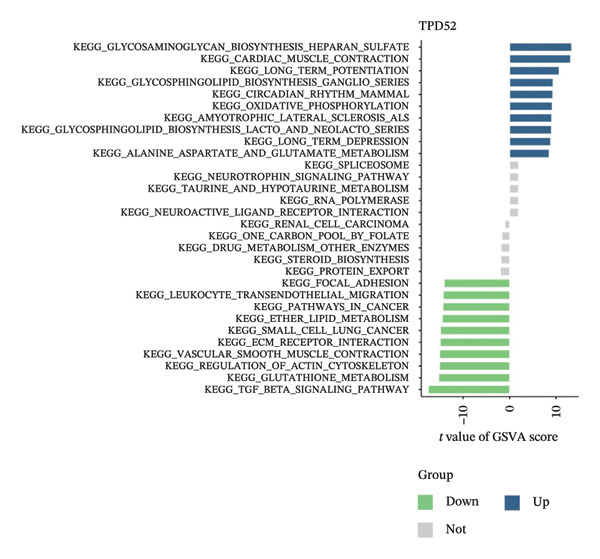
(h)

### 3.9. Experimental Validation

RT‐qPCR analyses confirmed a distinct PANoptosis expression profile in the NaIO_3_‐induced AMD model. Relative to saline‐treated controls, PON2 transcripts were significantly up‐regulated in model mice, whereas BNIP3, EPHB6, and TPD52 were each significantly down‐regulated (Figures 10(a), 10(b), 10(c), 10(d)). These coordinated changes validate our bioinformatic predictions and reinforce the involvement of PANoptosis dysregulation in retinal injury triggered by NaIO_3_.

FIGURE 10Experimental validation. (a‐b) mRNA expression levels of MMP‐9 and JNK‐2 in the blank group and model group. (c–e) Protein expression levels of MMP‐9 and JNK‐2 in the blank group and model group. A Student’s *t*‐test with *p* < 0.05 was used to determine statistical significance.  ^∗^ *p* < 0.05;  ^∗∗^ *p* < 0.01;  ^∗∗∗^ *p* < 0.001.

**Figure:**
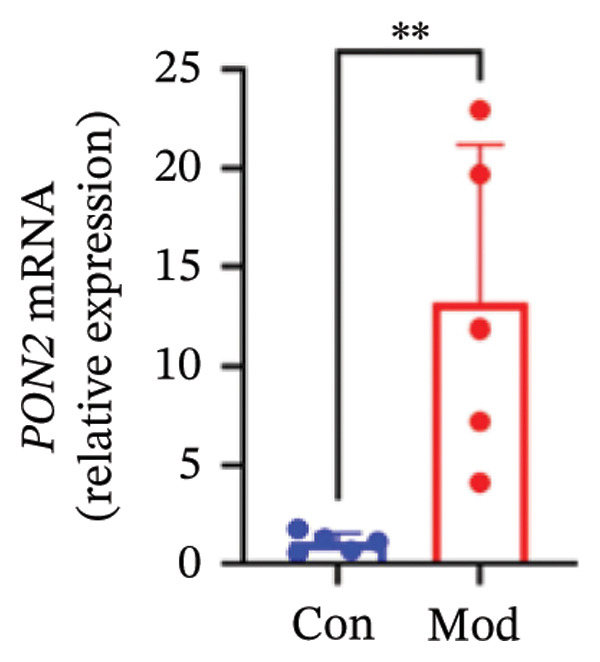
(a)

**Figure:**
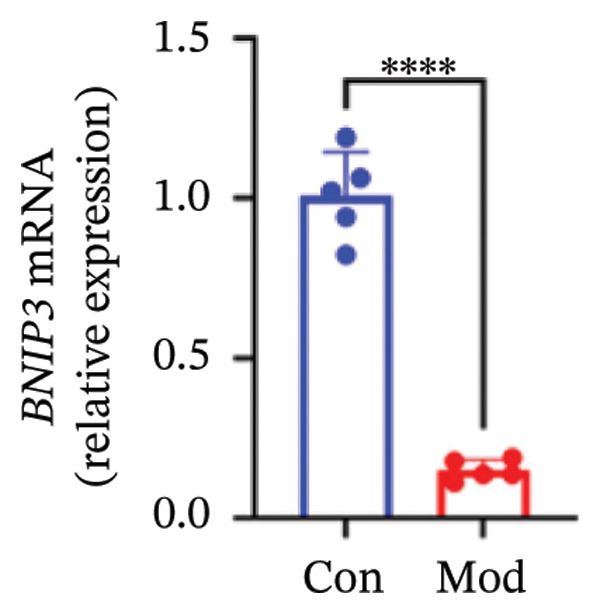
(b)

**Figure:**
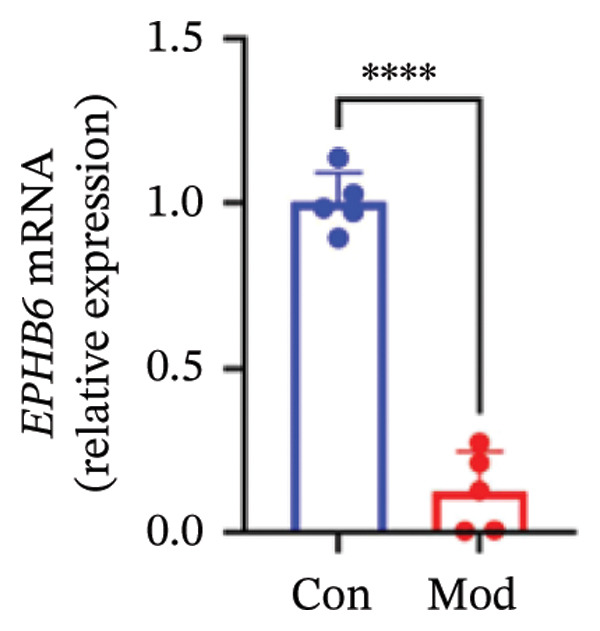
(c)

**Figure:**
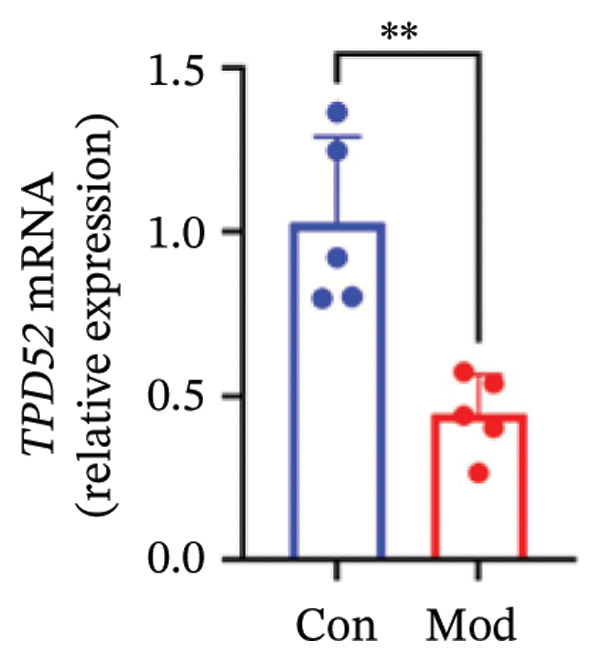
(d)

## 4. Discussion

AMD is a common ophthalmic disorder characterized by a chronic course and poor prognosis and remains a therapeutic challenge due to its complex and incompletely understood pathogenesis. The concept of PANoptosis was first introduced by Dr. Kanneganti’s group in 2019 [17] and has since emerged as a research hotspot in the fields of infection [18–20], autoimmune diseases [21], and cancer [22, 23]. Yan WT et al. demonstrated that in both in vivo and in vitro models of retinal neuronal ischemic injury, morphological alterations, changes in protein levels, and altered expression of key PANoptosome components (such as caspase‐1, NLRP3, and RIPK3) suggest the presence of PANoptosis‐like cell death in damaged retinal neurons [24]. Lin et al. reported that PANoptosis induced by inhibition of cysteine desulfurase (NFS1) enhances the antitumor efficacy of oxaliplatin‐based chemotherapy in colorectal cancer [18]. In an AMD model induced by Aβ1‐40 (a major component of drusen), Yuxia He et al. observed PANoptosis‐like cell death within the RPE–choroid complex. However, the role of PANoptosomes in RPE damage, as well as the underlying molecules and signaling pathways, remains to be elucidated [25].

By integrating single‐cell transcriptomic data, microarray datasets, and experimental validation, this study identified key genes and molecular mechanisms related to PANoptosis in AMD, and developed a disease risk prediction model. Using a nomogram model, the risk of AMD can be predicted based on the expression levels of these genes, providing a novel tool for clinical diagnosis. Moreover, calibration and decision curve analyses further confirmed the robustness and clinical utility of the model.

Our study identified four core genes (PON2, BNIP3, EPHB6, and TPD52) that may play crucial roles in the pathogenesis of AMD. Notably, Mendelian randomization analysis revealed a positive correlation between TPD52 and AMD, suggesting that TPD52 could be a potential therapeutic target. Furthermore, our findings highlighted the central role of macrophages in the pathological process of AMD. Macrophages are a key component of the retinal immune microenvironment, where classically activated M1 macrophages and alternatively activated M2 macrophages exert distinct functions in AMD pathology [26]. M1 macrophages are typically proinflammatory, releasing large quantities of cytokines such as IL‐1β and TNF‐α, thereby exacerbating retinal inflammation. In contrast, M2 macrophages are associated with anti‐inflammatory and tissue repair functions [27]. Using single‐cell RNA sequencing and UMAP‐based clustering, we identified six AMD‐associated cell subsets, including macrophages, with a significant increase in M1‐type macrophages observed in AMD patients. PANoptosis gene set scoring further revealed that macrophages had the highest scores among all annotated cell subsets, indicating a key role in the PANoptotic progression of AMD. Moreover, macrophages exhibited the highest communication frequency and strength with RPE cells, suggesting a potential synergistic role in the inflammatory and fibrotic processes of AMD. Aging, a well‐established major risk factor for AMD, leads to a reduction in RPE cell number, melanosome loss, and accumulation of lipofuscin [28], thereby exacerbating oxidative damage. Studies have shown that macrophages can extend dendritic processes into drusen in AMD, where macrophage‐mediated inflammation promotes the formation of subdrusen neovascularization, facilitating the progression from dry to wet AMD. Additionally, Abdoulaye et al. reported that aging alters macrophage expression of ATP‐binding cassette transporters ABCA1 and ABCG1, impairing their metabolic regulation and control of pathological angiogenesis, thereby intensifying inflammation in age‐related ocular diseases [29].

Intercellular communication plays a pivotal role in the pathological progression of AMD. Our study revealed that the TGFβ signaling pathway exerts critical regulatory effects within the pathological microenvironment of AMD, with highly cell type–specific activity, particularly in RPE cells and ECs. Epithelial‐to‐mesenchymal transition (EMT) and endothelial‐to‐mesenchymal transition (EndMT) are physiological processes essential for embryonic development, and in the eye, they play a key role in the pathogenesis of subretinal fibrosis in AMD [30]. Transforming growth factor‐β (TGFβ) is a central regulator of both EMT and EndMT and has been shown to critically influence AMD progression [31]. In constructing machine learning models, we employed algorithms including LASSO, SVM‐RFE, and RF to identify core genes and develop a nomogram‐based model for predicting AMD risk. This model demonstrated high predictive accuracy in both training and validation cohorts, with the RF model achieving an AUC of 0.932, indicating strong clinical application potential. SHAP analysis further elucidated the contribution of each core gene to model performance, offering novel insights into their biological roles in AMD. The four core genes identified (PON2, BNIP3, EPHB6, and TPD52) show significant potential in AMD diagnosis and risk prediction. PON2 (paraoxonase 2), a member of the paraoxonase family, exhibits antioxidative, anti‐inflammatory, and anti‐apoptotic properties and is primarily involved in the regulation of intracellular oxidative stress and lipid metabolism. The PON family is implicated in the pathogenesis of various inflammatory diseases, including Alzheimer’s disease, atherosclerosis, Parkinson’s disease, and cancer [32]. PON2 deficiency exacerbates mitochondrial dysfunction (e.g., reduced complex I/III activity and impaired ATP synthesis), leading to excessive ROS accumulation and lipid peroxidation, which in turn activates MAPK pathways (e.g., JNK/p38) and promotes PANoptosis in RPE cells. Additionally, PON2 deficiency aggravates mitochondrial oxidative stress, as evidenced by elevated superoxide production, increased lipid peroxidation, and decreased glutathione levels [33, 34]. Sreekumar PG et al. also demonstrated that PON2‐deficient mice exhibit increased retinal apoptosis and functional and structural damage, rendering them more susceptible to RPE mitochondrial dysfunction and retinal degeneration [35]. YE Y et al. found that PON2 expression is depleted in Ang II–stimulated AC16 cells, and PON2 may mediate CANX/NOX4 signaling to suppress oxidative stress, inflammation, hypertrophy, and injury in these cells [36]. BNIP3 (BCL2/adenovirus E1B 19 kDa–interacting protein 3) is a key pro‐apoptotic protein belonging to the BH3‐only subfamily of the BCL‐2 protein family. It interacts with other BCL‐2 family members (such as BCL‐2 and BCL‐XL) to regulate mitochondrial outer membrane permeability, thereby inducing apoptosis [37]. BNIP3 is widely expressed in various tissues and cell types, and its primary functions involve the regulation of apoptosis, autophagy, and mitochondrial homeostasis, particularly under conditions of hypoxia, oxidative stress, and metabolic disturbance. In addition, BNIP3 promotes mitophagy to eliminate damaged mitochondria, thereby maintaining cellular energy homeostasis. BNIP3 plays dual roles in multiple diseases. In acute kidney injury, Tang CY et al. demonstrated that BNIP3 exerts a protective effect in renal tubular cells by regulating mitophagy. Loss of BNIP3 function inhibits mitophagy, resulting in inefficient clearance of damaged mitochondria, excessive accumulation of reactive oxygen species (ROS), and aggravated cell death [38]. EPHB6 (Eph receptor B6) is an important member of the Eph receptor tyrosine kinase family and functions as a transmembrane protein. Its biological functions are mainly mediated through binding to ephrin‐B family ligands. Unlike other Eph receptors, EPHB6 lacks a functional kinase domain, and its signaling depends on interactions with other Eph receptors or downstream effectors. EPHB6 plays critical roles in cell adhesion, migration, differentiation, and tissue development and is particularly involved in the regulation of neural, immune, and tumor microenvironments. In a study investigating the regulatory role of EPHB6 in lung cancer metastasis, loss of EPHB6 expression was associated with increased metastatic frequency in early‐stage non‐small cell lung cancer (NSCLC), suggesting that EPHB6 may directly suppress cancer metastasis [39]. TPD52 (tumor protein D52) is a broadly expressed protein belonging to the TPD52 family and is involved in various biological processes, including cell proliferation, apoptosis, vesicle trafficking, and signal transduction. As its name suggests, TPD52 is highly expressed in numerous tumor types, and targeted regulation of TPD52 expression has been shown to exert antitumor effects [40, 41]. However, the role of TPD52 in nontumor diseases remains largely unexplored and warrants further investigation.

This study is the first to explore the role of PANoptosis in AMD, thereby introducing a novel research direction. As a newly defined form of PCD, PANoptosis remains underexplored in the context of AMD, underscoring the innovation of this work. The study employed a variety of bioinformatics tools and machine learning algorithms (e.g., LASSO, SVM‐RFE, and RF) to ensure the robustness and accuracy of the results. Moreover, the application of advanced approaches such as Mendelian randomization analysis and SHAP‐based model interpretation added further depth and breadth to the investigation. Core genes associated with AMD (e.g., PON2, BNIP3, EPHB6, and TPD52) were identified and incorporated into a predictive model, offering potential biomarkers and therapeutic targets for early diagnosis and intervention. Nonetheless, several limitations should be acknowledged. Although the functions of the four core genes were validated at the mRNA level using qPCR, no further protein‐level investigations were conducted. In‐depth experimental validation of the specific functional mechanisms of these genes is also lacking. For example, gene knockout or overexpression experiments could help clarify their specific roles in AMD. Additionally, while this study highlights the critical involvement of macrophages and RPE cells in AMD, their functional roles have yet to be experimentally validated, such as via in vitro co‐culture or organoid models. A further limitation relates to clinical heterogeneity and disease‐subtype mismatch between datasets and experimental models. The bulk transcriptomic cohort includes AMD samples spanning early AMD, geographic atrophy, and neovascular subtypes, whereas our NaIO_3_‐induced retinal degeneration model mainly recapitulates RPE injury/atrophy characteristic of dry (atrophic) AMD and does not model choroidal neovascularization. Consequently, although the identified PANoptosis‐related genes distinguish AMD from normal eyes, our current experimental validation is most directly relevant to dry/atrophic AMD, and additional studies in independent neovascular AMD cohorts are required to confirm their diagnostic and mechanistic relevance for wet AMD. Future work should focus on expanding the sample size, investigating the mechanistic roles of core genes in greater depth, and performing external validation to enhance the clinical utility of the predictive model.

## Author Contributions

J.L. and Y.M. conducted the bioinformatics research. J.L., Y.M., and M.H. designed the research methods. Q.Z., A.W., Q.G., and Q.T. analyzed the data. Y.M. and J.L. wrote the manuscript. B.H. led the entire research project and reviewed and approved the final version of the manuscript.

## Funding

This work was supported by the National Natural Science Foundation of China (grant number 82104935); Jiangxi Natural Science Foundation (grant number 20252BAC240450); Jiangxi University of Chinese Medicine Graduate Student Innovation Special Fund (translated name) (grant number XJ‐S202468); Science and Technology Plan of the Jiangxi Provincial Administration of Traditional Chinese Medicine (translated name) (grant number 2024B0134); and Jiangxi Province Science Education Association (translated name) (grant number 2025KXJYS438).

## Disclosure

All authors have read and approved the final manuscript.

## Ethics Statement

Experimental animals were housed in the SPF‐level laboratory at Jiangxi University of Chinese Medicine. The handling of animals during the experiment was approved by the Jiangxi University of Traditional Chinese Medicine Laboratory Animal Science and Technology Center (Facility License No. SYXK (Gan) 2022‐0002).

## Conflicts of Interest

The authors declare no conflicts of interest.

## Supporting Information

Additional supporting information can be found online in the Supporting Information section.

## Supporting information


**Supporting Information 1** Supporting File 1: PANoptosis‐related genes.

**Supporting Information 2** Supporting File 2: (A) Dataset GSE188280 before quality control. (B) Dataset GSE188280 after quality control. (C) 2000 highly variable genes.

**Supporting Information 3** Supporting File 3: Cell cluster annotation file.

**Supporting Information 4** Supporting File 4: (A‐C) UMAP visualization including all cells, colored by group (AMD vs. Control) and by sample.

## Data Availability

The datasets GSE29801 and GSE188280 for this study can be found at https://www.ncbi.nlm.nih.gov/geo/. The data supporting the findings of this study are available from the corresponding author upon a reasonable request.
