# Supplementary material for: PANoptosis‐Related Diagnostic Biomarkers in Non‐Neovascular Age‐Related Macular Degeneration: An Integrative Transcriptomic and Experimental Study
Source: Genet Res (Camb). 2026 Feb 13;2026:8903808. doi: 10.1155/genr/8903808 (PMC12905011; doi:10.1155/genr/8903808)
Supplement: Supplementary file 2 — Supporting Information 2 Supporting File 2: (A) Dataset GSE188280 before quality control. (B) Dataset GSE188280 after quality control. (C) 2000 highly variable genes. [file GENR-2026-8903808-s004.pdf]

A

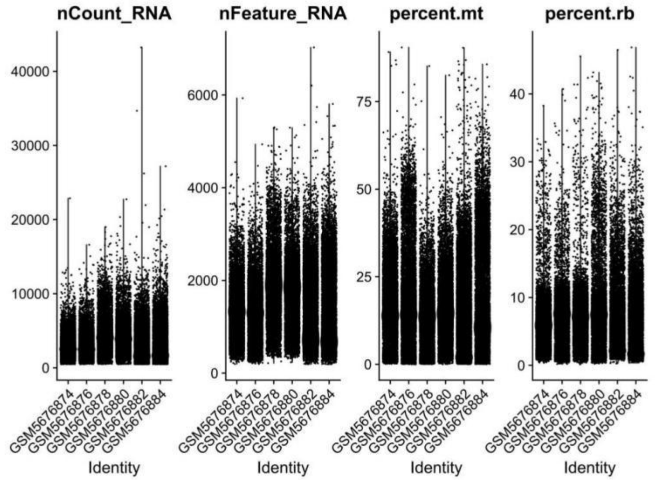

B

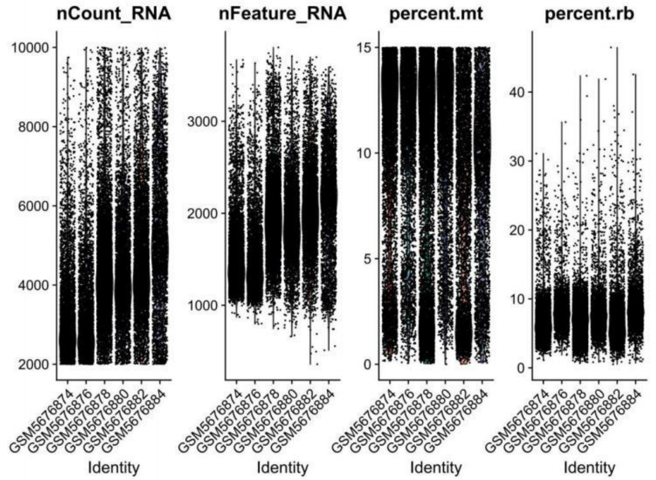

C

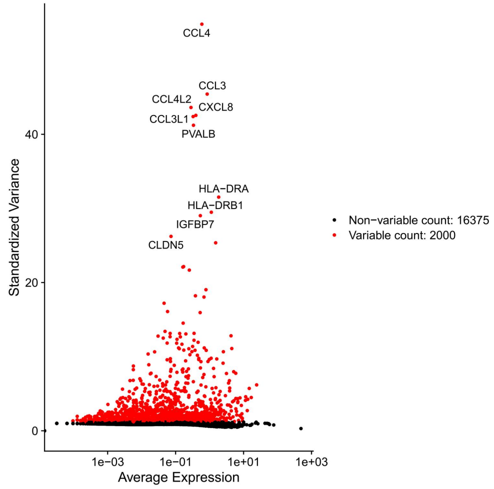

Supplementary File 2: (A) Dataset GSE188280 before quality control. (B) Dataset GSE188280 after quality control. (C) 2,000 highly variable genes.
