# Supplementary material for: PANoptosis‐Related Diagnostic Biomarkers in Non‐Neovascular Age‐Related Macular Degeneration: An Integrative Transcriptomic and Experimental Study
Source: Genet Res (Camb). 2026 Feb 13;2026:8903808. doi: 10.1155/genr/8903808 (PMC12905011; doi:10.1155/genr/8903808)
Supplement: Supplementary file 4 — Supporting Information 4 Supporting File 4: (A‐C) UMAP visualization including all cells, colored by group (AMD vs. Control) and by sample. [file GENR-2026-8903808-s002.pdf]

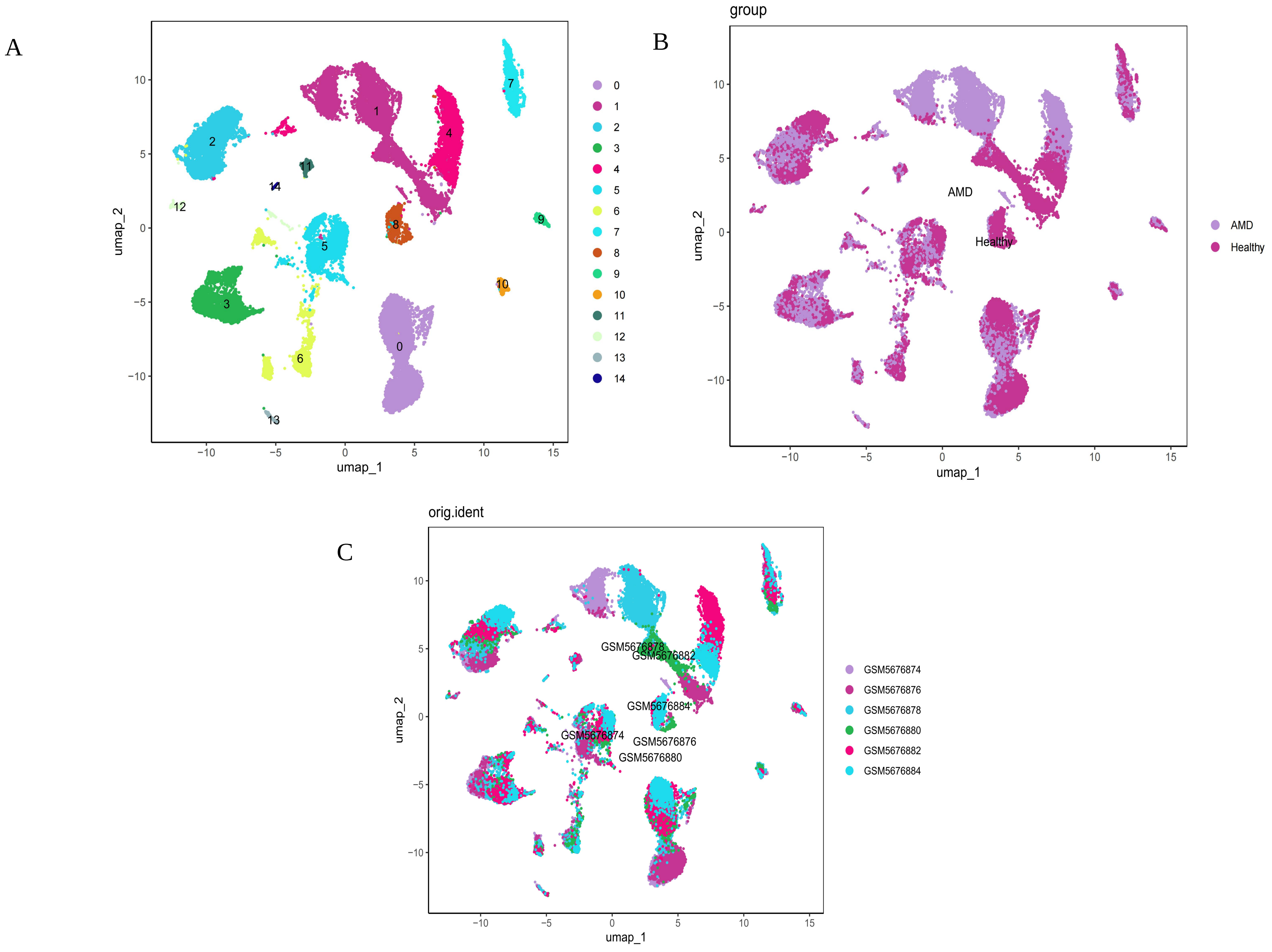

Supplementary Figure 4：(A-C) UMAP visualisation including all cells, coloured by group (AMD vs Control) and by sample
